# Psychological Interventions for the Fear of Public Speaking: A Meta-Analysis

**DOI:** 10.3389/fpsyg.2019.00488

**Published:** 2019-03-15

**Authors:** Omid V. Ebrahimi, Ståle Pallesen, Robin M. F. Kenter, Tine Nordgreen

**Affiliations:** ^1^Department of Clinical Psychology, Faculty of Psychology, University of Bergen, Bergen, Norway; ^2^Division of Psychiatry, Haukeland University Hospital, Bergen, Norway

**Keywords:** fear of public speaking, public speaking anxiety, social anxiety disorder, meta-analysis, systematic review, psychological treatment, randomized controlled trial

## Abstract

**Background:** Fear of Public Speaking (FoPS) or public speaking anxiety is a type of social anxiety and the single most commonly feared situation in the population. FoPS is disabling with negative occupational, academic, and social consequences, reported by up to one third of the population. FoPS in adolescence and adulthood is associated with an increased risk of developing generalized social anxiety disorder with further impairments. Since the last review on FoPS, a significant number of randomized controlled trials (RCTs) have been conducted assessing the effects of novel interventions with innovative modes of delivery.

**Objectives:** The objectives of the present meta-analysis are to (1) examine the short and long-term effects of psychological interventions aimed at FoPS on FoPS and generalized social anxiety; (2) assess whether differences exist between technology-assisted modes of delivery (e.g., Internet-delivered therapies) and more traditional modes of delivering treatment (e.g., face-to-face therapies); (3) investigate whether differences in effect exist between theoretical frameworks; (4) inspect the differences in effect size between self-report measures and other measures (i.e., physiological and behavioral); (5) examine the effects of psychological interventions aimed at FoPS on secondary outcome measures (e.g., depression); and (6) investigate whether a “sleeper effect” is present for psychological interventions for FoPS and generalized social anxiety.

**Methods:** The study investigates the effects of psychological interventions for FoPS through a quantitative meta-analysis of RCTs, using a random-effects model.

**Results:** A total of 30 RCTs with 1,355 participants were included through systematic searches of PsycINFO, MEDLINE, Web of Science, and Cochrane Library. The majority of the studies investigated the effects of cognitive or behavioral interventions. Nearly half of the studies used active control groups (e.g., attention placebo), whereas the other half used passive (e.g., waitlist) controls. The overall effect of psychological interventions for FoPS across 62 interventions was 0.74 (Hedges *g*; 95% CI: 0.61–0.87) with low to moderate heterogeneity. No difference in effect was found across theoretical frameworks. The effects based on self-report measures were larger compared to physiological and behavioral outcomes. Effects were robust against both active and passive control groups. Furthermore, psychological interventions for FoPS had a small to moderate effect on generalized social anxiety disorder (*g* = 0.35; 95% CI: 0.22–0.48). The effect of psychological interventions aimed at FoPS at follow-up was large (*g* = 1.11, 95% CI: 0.90–1.31) and moderate to large for generalized social anxiety (*g* = 0.70, 95% CI: 0.59–0.80). A sleeper effect was found for cognitive and behavioral interventions, indicating that patients continued to improve after treatment termination. There were some indications of publication bias.

**Conclusions:** Psychological interventions are effective in reducing FoPS. Interventions using technology-assisted modes of delivery are equally effective as traditional face-to-face interventions in reducing FoPS. This finding highlights an opportunity to increase access to evidence-based treatments through technology-delivered interventions, which can be implemented at schools, in primary care and specialist mental health care. Moreover, psychological interventions aimed at FoPS have an effect on generalized social anxiety. Further implications are discussed.

## Introduction

Fear of Public Speaking (FoPS), also referred to as public speaking anxiety, is a costly (Lépine, 2002) and disabling fear (Blöte et al., 2009), with prevalence rates ranging from 21 (Pollard and Henderson, 1988) to 33% (Stein et al., 1996; D'El Rey and Pacini, 2005) in community samples. FoPS has been reported as the single most commonly feared situation in both university and community samples (Pollard and Henderson, 1988; Holt et al., 1992; Stein et al., 1996; Tillfors and Furmark, 2007). Additionally, FoPS is a fear that almost always has its onset in adolescence (Wittchen and Fehm, 2003).

FoPS has consistently been described as a specific type of social anxiety disorder (SAD; Heimberg et al., 1993; Blöte et al., 2009). SAD is the most common anxiety disorder with a lifetime prevalence of 12.1% (Stein and Stein, 2008). SAD is characterized by a considerable fear or anxiety in social interaction or performance situations in which the individual is exposed to unfamiliar people or possible scrutiny by others (American Psychiatric Association, 2013). SAD is highly impairing, disabling, reduces quality of life, has negative scholastic, occupational and social consequences (Stein and Kean, 2000; Fehm et al., 2005), and has great societal costs both directly and indirectly (e.g., through absenteeism from work; Dams et al., 2017). There are two broad subgroups of social anxiety. One of these involves individuals with both interaction and performance anxiety, referred to as generalized social anxiety disorder (Blöte et al., 2009; Bögels et al., 2010). The other involves individuals with only performance anxiety, where FoPS is its most common form (Blöte et al., 2009; Bögels et al., 2010). This distinction is in accordance with the DSM-5 (American Psychiatric Association, 2013) including a “performance only”-specifier within the social anxiety diagnosis for individuals whom have the specific fear of performing in front of others, such as FoPS.

Around half of the adolescents with FoPS (Hofmann et al., 1999) and half of the adults with FoPS (Blöte et al., 2009) develop generalized social anxiety. A prospective follow-back study by Gregory et al. (2007) reports that both anxiety disorders and specific phobias in adulthood are frequently preceded by phobias in the adolescent years. In addition to the impairing consequences of FoPS on its own (Pollard and Henderson, 1988; Fehm et al., 2005), untreated FoPS in both adolescent and adult years is associated with later generalized social anxiety disorder (Wittchen and Fehm, 2003; Blöte et al., 2009) with further disabling consequences. The high prevalence and negative consequences of FoPS, taken together with the fact that FoPS increases the risk of a more generalized SAD, makes it important to update the knowledge base on effective interventions for FoPS.

### Rationale

Over the past decades, several randomized controlled trials (RCTs) have been conducted to investigate the effect of psychological interventions for FoPS (e.g., Newman et al., 1994; Harris et al., 2002; Anderson et al., 2013; McNally et al., 2013; Homer et al., 2016). These trials comprise a wide range of psychological interventions, where most are based on the cognitive and/or behavioral family of therapies (e.g., Karst and Trexler, 1970; Newman et al., 1994). Still, psychological interventions within the traditions of psychodynamic therapies such as visualization therapy based on psychosynthesis (Ayres, 1995) and insight therapy (Meichenbaum et al., 1971) have been utilized. Different modes of delivery of interventions have also been investigated, such as Internet-delivered therapies (e.g., Botella et al., 2010) and interventions that implement virtual reality scenarios as a method of exposure (e.g., Anderson et al., 2013).

The last and only meta-analysis conducted solely on FoPS (Allen et al., 1989) is nearly 30 years old and is exclusively based on self-report measures, in addition to including uncontrolled and non-randomized studies. The fact that the results are only based on self-report is problematic, as previous studies on social anxiety (e.g., Heimberg et al., 1990) have found that the effects of interventions assessed through self-report measures are larger as compared to other types of measures (i.e., physiological and behavioral measures). Additionally, the inclusion of uncontrolled and non-randomized studies in the meta-analysis by Allen et al. (1989) brings uncertainty to its results, as it hinders estimation of the effects of psychological interventions for FoPS compared to control groups, presenting fundamental problems concerning the elimination of confounding variables, maturation, spontaneous recovery, and regression to the mean. A more recent meta-analysis by Acarturk et al. (2009) examined the effects on psychological treatment of social anxiety disorder. This meta-analysis included seven studies that examined the effects of FoPS interventions, but in their analysis the authors did not examine the effects of psychological interventions for FoPS specifically, but rather looked at the difference for generalized social anxiety disorder and specific social anxiety disorder. Thus, the efficacy of psychological interventions for FoPS exclusively based on RCTs remains unknown to date. Furthermore, several new RCTs have been conducted since the meta-analysis by Allen et al. (1989) and Acarturk et al. (2009), utilizing interventions with novel modes of delivery that are yet to be examined in a meta-analysis. The present study will be the first to assess and compare the effects of different modes of delivery for interventions for FoPS, such as technology-delivered interventions (e.g., Internet-delivered interventions and virtual reality-based interventions) and face-to-face interventions. The present meta-analysis is furthermore the first meta-analysis on FoPS that examines changes in effect after treatment termination, also referred to as the “sleeper effect” (Flückiger et al., 2015). Taken together, there is a need for updated knowledge on effective psychological interventions for FoPS.

### Objectives

The present meta-analysis will provide synthetized information about treatment of FoPS to clinicians and researchers. Specifically, the effects of psychological interventions for FoPS will be examined across age-groups, modes of delivery and theoretical orientation. The meta-analysis includes RCTs with a control group, regardless of type (e.g., attention placebo or waitlist control). The effect of FoPS interventions on generalized social anxiety disorder will be investigated. We will also elucidate the effect of psychological interventions for FoPS for all types of outcome measures (i.e., physiological, self-report, and behavioral). The present meta-analysis will furthermore provide an evaluation of newer modes of treatment delivery for FoPS (e.g., Harris et al., 2002; Botella et al., 2010; Anderson et al., 2013), which is of practical significance for clinicians in guiding treatment selection.

### Research Aims

The present study had several aims, the first of which was to examine the overall effect of psychological interventions for FoPS. Our second aim was to evaluate the long-term effects of such psychological interventions for FoPS. The third aim was to investigate whether there is a difference between technology-assisted modes of delivery of interventions for FoPS (i.e., Internet-delivered therapies, virtual reality exposure therapies and computerized interventions) vs. traditional face-to-face interventions. The fourth aim was to examine whether there is a difference between cognitive and/or behavioral interventions compared to other therapeutic frameworks (e.g., visualization and insight therapy). The fifth aim was to investigate if there would be difference in effect size between self-report measures as compared to other outcome measures such as behavioral or observational measures (assessing overt signs of anxiety) and physiological measures. The sixth aim was to investigate whether psychological interventions of FoPS have short- and long-term effects on generalized social anxiety. Provided sufficient data were available, another aim of this study was to examine the effects of psychological interventions for FoPS on secondary outcome measures of depression, satisfaction with treatment, outcome expectancy and treatment credibility. Finally, changes in outcomes after treatment termination were investigated.

## Methods

The meta-analysis was prepared in accordance with the guidelines of the Preferred Reporting Items for Systematic Reviews and Meta-analyses (PRISMA; Moher et al., 2009) and the Meta-Analysis Reporting Standards (MARS; American Psychological Association, 2008).

### Systematic Review Protocol

The pre-registered protocol of this study can be found in the PROSPERO International Prospective Register of Systematic Reviews (https://www.crd.york.ac.uk/prospero/display_record.php?RecordID=60702). The search strategy, inclusion and exclusion criteria, data extraction, risk of bias assessment, strategy for data synthesis and subgroup analyses adhered to the preregistered study protocol.

### Participants, Interventions, Comparators

The present meta-analysis included (a) randomized controlled trials in which the effects of (b) psychological interventions for (c) FoPS were assessed (d) across any age group. Participants were required to (e) have been identified as having a problem with FoPS either through a diagnosis of social anxiety with public speaking as their primary fear; through scoring above a certain cut-off point on an instrument measuring FoPS; through evidence of elevated scores (e.g., one standard deviation above the mean) on an instrument assessing FoPS; or through self-identification of FoPS as an impairing problem. Furthermore, studies were included if (f) the intervention group was compared to a control group of any of the following kind: sham, or attention placebo, treatment as usual or minimal contact, waiting list control or no treatment control. For studies with two or more control conditions, the control group selected for the calculation of effect size was chosen, as a conservative approach, in the order specified above, with active control groups (e.g., attention placebo) being preferred over passive controls (e.g., waiting list control).

Psychological interventions were defined as interventions designed to decrease psychological symptoms, distress, and maladaptive behavior or designed to improve prosocial and adaptive functioning using interpersonal interactions, counseling, or activities following a specific treatment plan (Garfield, 1980; Walrond-Skinner, 1986).

Studies were excluded if they (a) failed to meet the inclusion criteria described above, (b) were duplicate studies, (c) were studies in a language other than English, German, Dutch, Norwegian, Danish or Swedish, (d) were studies that did not provide sufficient information to calculate effect sizes, (e) were studies where the participant had been identified as having a problem with generalized social anxiety (not FoPS specifically), and (f) were studies in which participants were identified as having a problem with communication apprehension generally (not FoPS specifically). If a study did not provide sufficient information for the calculation of effect sizes, the study authors were contacted in an attempt to acquire the necessary data to include the study.

### Search Strategy

The search strategy was constructed by three of the authors (OE, TN, and RK) of the present study through identification and discussion of relevant keywords, accompanied by preliminary searches identifying further relevant keywords to increase search sensitivity. Relevant studies were primarily identified through systematic searches in major bibliographical databases, including PsycINFO, MEDLINE, Web of Science, and the Cochrane Library. The last search was conducted by the first author on January 19, 2018. No restriction was set concerning how far the search could go back in time in order to include all relevant studies. In the searches, different combinations of words indicative of FoPS, speech phobia, fear of presenting and communication apprehension were combined with words like intervention, treatment, psychotherapy, and related words. In an attempt to increase the sensitivity of the searches, we did not further limit the searches by searching for terms indicative of RCTs. Both text words and keywords were utilized. An overview of the keywords and search strategy can be found in “Supplemental Material A.” Furthermore, database searches were supplemented by manually searching already published, relevant, systematic reviews, and meta-analyses (Allen et al., 1989; Pull, 2012). Reference lists of included studies in the meta-analysis were also searched.

### Data Extraction

Two independent researchers screened all titles and abstracts of the retrieved references for eligibility against the inclusion criteria. Disagreements were resolved in consultation with a third independent, senior researcher. The first author obtained the full text of eligible studies before two independent researchers assessed them for inclusion.

Several aspects of the included studies were coded in line with the Cochrane Review data extraction template in addition to coding further aspects of the studies that we deemed relevant through the pilot testing of our data extraction procedure. Where available, the following data were extracted: Study characteristics (e.g., year, country, design, sample size, type of control group, and trial duration); sample characteristics (e.g., age, sex, sample description, description of comorbidities, and ethnicity); intervention characteristics (e.g., intervention name, details, number of sessions, attrition, format, mode of delivery, and theoretical framework); and outcome characteristics for both FoPS and social anxiety (e.g., name of outcome instrument, type of measurement, time points, and scores). Measures of FoPS and social anxiety served as the primary outcome measures in the present study. When available, we also coded measures of depression, satisfaction with treatment, outcome expectancy and treatment credibility, which served as secondary outcome measures. We coded the type of control group used in the study as either active (e.g., attention placebo) or passive (e.g., waiting list or no treatment control). Sample description was coded as diagnostic in cases where individuals were identified with a formal diagnosis of social anxiety with FoPS as their primary fear, and as non-diagnostic where the individuals were identified through cut-offs, elevated scores, or had self-identified as having an impairing problem with FoPS. The format of the interventions was coded as individual, group or self-help. Mode of delivery was coded as technology-delivered (Internet-delivered, virtual reality based, computerized, or video-based interventions) and non-technological (traditional) interventions (face to face, telephone-based or self-help). Coding the theoretical framework of the different interventions was challenging as most of the interventions included a mixture of different cognitive and behavioral components. We therefore, in accordance with a previous meta-analysis, followed the example of Cuijpers et al. (2014) and coded the intervention belonging to the broad family of cognitive and/or behavioral interventions if it included at least one of the following components; exposure, cognitive restructuring, relaxation, biofeedback, and problem solving. Interventions not in this category were, once again in accordance with Cuijpers et al. (2014), coded as “other” interventions, representing non-cognitive or non-behavioral interventions including visualization therapy based on psychosynthesis, eye movement desensitization and reprocessing (EMDR), exercise, and insight therapy.

### Risk of Bias Assessment

In agreement with prior meta-analyses (e.g., Cuijpers et al., 2014, 2016), we used four criteria of the “Risk of Bias” assessment tool developed by the Cochrane Collaboration (Higgins et al., 2011) to assess the sources of bias in the included RCTs. Two independent researchers rated the following domains of bias: (1) adequate generation of allocation sequence (selection bias); (2) concealment of allocation to conditions (selection bias); (3) prevention of knowledge of the allocation intervention or blinding of outcome assessors (detection bias); and (4) dealing with incomplete outcome data (attrition bias). Disagreements were resolved through discussion with a third independent senior researcher, in addition to contacting the study authors in cases of insufficient reporting for clarification. In the present meta-analysis, we judged a randomized controlled trial to be high in terms of risk of attrition bias if the dropout rate of the intervention group was higher than 10%, or if there was a considerable discrepancy in drop-out rates between the intervention and comparing conditions as defined by the Cochrane risk of bias tool guidelines (Higgins et al., 2011). Based on the abovementioned sources of bias and in line with the Cochrane Handbook for Systematic Reviews of Interventions (Higgins and Green, 2011), we categorized each domain of bias within a study to have low, high or unclear risk, respectively. In accordance with previous meta-analyses (e.g., Cuijpers et al., 2014), we coded outcomes solely based on self-report measures as “SR” in the risk of bias domain concerning blinding of outcome assessors (detection bias).

### Meta-Analyses

To calculate the effects of psychological interventions for FoPS, the effect size demonstrating the difference between the intervention and control group at post-treatment was calculated using Cohen's *d* (standardized mean difference). These were calculated by subtracting the mean score of the psychological intervention group at post-treatment from the mean score of the control group at post-treatment, before dividing the result by the pooled standard deviation formed by the two groups, and adjusting the effect size to account for small sample bias in accordance with the procedures advised by Hedges and Olkin (1985; Hedges' *g*) When available, we based our comparisons on intention-to-treat data for the calculation of effect sizes. If intention-to-treat data was unavailable, we based our calculations on completers-only data. Effect sizes (*g*) of 0.2 are identified as small, effect sizes of 0.5 are considered moderate, whereas effect sizes of 0.8 are referred to as large (Cohen, 1988). If means and standard deviations were not reported, we used the accompanying procedures in the Comprehensive Meta-analysis Software (version 3.3.070; CMA) to calculate effect sizes based on other statistics (e.g., *t, p*, and *F* value). The effect size for generalized social anxiety disorder was calculated in the same manner as described above.

The long-term effects of psychological interventions on FoPS were calculated as explained above, based on comparisons between the intervention group and a control group (between-group comparison). If a control group was not available at the follow-up assessment, we calculated effect sizes indicative of improvement from baseline to follow-up for the treatment condition (within-group comparisons). Since the values at baseline and follow-up are not independent of each other, a conservative correlation between baseline and follow-up score of *r* = 0.70 was assumed, following procedures used in other meta-analyses (Grossman et al., 2004; Ledesma and Kumano, 2009; Cuijpers et al., 2016). The same procedures were employed to investigate whether psychological interventions aimed at FoPS had long-term effects on the more generalized form of social anxiety.

Furthermore, we conducted two meta-analyses to examine the changes in effect after treatment termination, also referred to as the “sleeper effect” (e.g., Flückiger et al., 2015). One of these meta-analyses concerned the changes on FoPS, more specifically from post-treatment to follow-up. The average effect size was calculated based on within-group comparisons (post-treatment to follow-up). As the values at post-treatment and follow-up scores are not independent of each other, a conservative correlation between post-treatment and follow-up score of *r* = 0.70 was assumed, following procedures used in other meta-analyses (Grossman et al., 2004; Ledesma and Kumano, 2009; Cuijpers et al., 2016). The second meta-analysis concerned generalized social anxiety outcomes from post-treatment to follow-up. Its effect size was calculated in the same manner as described above. We thus conducted six meta-analyses, two investigating the post-treatment effects of psychological interventions aimed at FoPS on FoPS and generalized social anxiety, respectively, two meta-analysis investigating the long-term effects of psychological interventions on the two same parameters, and two meta-analyses examining the changes in effect after treatment termination on the two parameters.

Some studies report multiple comparisons, where two or more psychological interventions were compared to the same control group. A potential consequence of such multiple comparisons is an artificial reduction of heterogeneity, which in turn can affect the overall effect size. In order to take this into account, we followed the procedure of Cuijpers et al. (2014) comprising separate analyses that include only the smallest and the largest effect size from each study, respectively.

As the standardized mean difference (Hedges' *g*) can be difficult to interpret from a clinical viewpoint, we transformed this into numbers-needed-to-treat (NNT) following the procedure and formulae provided by Kraemer and Kupfer (2006). The NNT can be described as the number of patients that would have to be treated in order to generate one additional positive outcome (Laupacis et al., 1988).

Furthermore, the present study differentiated between three categories of outcome measures in the calculation of effect sizes: (a) self-report measures; (b) behavioral or observational measures (e.g., measuring overt signs of anxiety); and (c) physiological measures (e.g., heart rate or pulse rate). If the effect of an intervention was assessed by more than one outcome measure, we pooled all relevant instruments to provide one average effect size rather than imputing effect sizes for each outcome measure, as suggested by Borenstein et al. (2009b). This is a conservative approach as it somewhat overestimates the study variance resulting in wider confidence intervals compared to approaches taking the independence of the outcomes into consideration. The current approach was used as the included studies in general did not report the correlation coefficient between the different outcome measures. The percentage of outcome measures that was not based on self-report was coded for the purpose of moderation analyses.

CMA, version 3.3 was used for calculation of the pooled mean effect sizes. The random-effects pooling model was utilized in all analyses to account for the expected heterogeneity. The statistical assumptions underlying the random-effects model imply that the included studies stem from populations that vary systematically. Taking this into consideration, the difference in effect size results not only from random error within studies (as the fixed effects model assumes) but also from true variation in effect size, as studies are assumed to represent a different population of studies.

Heterogeneity of effect sizes was assessed by the Q-statistic and the *I*^2^-statistic. The latter is an indicator of variance (0–100%) between studies that is due to heterogeneity. Values equal to and lower than 25% are considered low; values of 50% as moderate, and values equal to and higher than 75% as high heterogeneity (Higgins et al., 2003).

### Meta-Regression Analyses

In cases of significant heterogeneity, meta-regression analyses would be conducted to test whether a priori selected moderator variables could explain the heterogeneity. Maintaining a reasonable ratio of single effect sizes/moderators implied restrictions on the number of moderators that could be analyzed in the present meta-analyses. In line with this, a limited number of a priori selected moderators were analyzed. The effect size was used as the dependent measure in these multivariate meta-regression analyses which were conducted in CMA.

For the first meta-analysis in the present study concerning the effects of psychological interventions on FoPS, the following moderator variables were pre-selected: (1) technology-delivered vs. non-technological interventions; (2) theoretical framework (cognitive or behavioral vs. other interventions); (3) percentage of measures other than self-report; and (4) type of control group (active control groups including attention placebo vs. passive no-treatment or waiting list groups).

For the second meta-analysis examining the effects of psychological interventions aimed at FoPS on generalized social anxiety disorder, we pre-selected the latter three mentioned moderator variables above.

For the meta-analyses concerning the long-term effects of psychological interventions on FoPS and generalized social anxiety, respectively, the pre-selected moderator variables were (1) time from post-treatment to follow-up, and (2) whether the effect size at follow-up was calculated within-groups (comparing pre-treatment scores to follow-up scores) or between-groups (comparing intervention group to control group). Meta-regression analyses would only be conducted in cases of significant heterogeneity, indicated by a significant Q-statistic.

As the meta-regressions include all moderators in the same analysis, potential dependence between moderators was controlled for. All the included studies provided information of all moderators, hence, the potential dependence within each study was controlled for. Furthermore, for each single effect size, only one specific level of each moderator was coded. Hence dependencies between categories/levels of the same moderator was not an issue.

### Publication Bias

Furthermore, publication bias was examined through inspecting the funnel plot on primary outcome measures and by following the procedures suggested by Duval and Tweedie's trim and fill procedure (Duval and Tweedie, 2000). This procedure provides an adjusted estimate of the effect size after accounting for publication bias. The present meta-analysis also calculated Orwin's fail-safe *N* (Orwin, 1983), a measure quantifying the amount of studies that would be needed to bring the observed effect size (calculated in Hedges' *g*) of the current meta-analysis down to a chosen “trivial” effect size with less importance. We set this “trivial” effect size to a value of 0.2 Hedges' *g*, since a value of 0.2 represents a small effect (Cohen, 1988). This test was conducted in an attempt to take into account the file-drawer problem (Rosenthal, 1979).

## Results

### Selection and Inclusion of Studies

**Figure 1:**
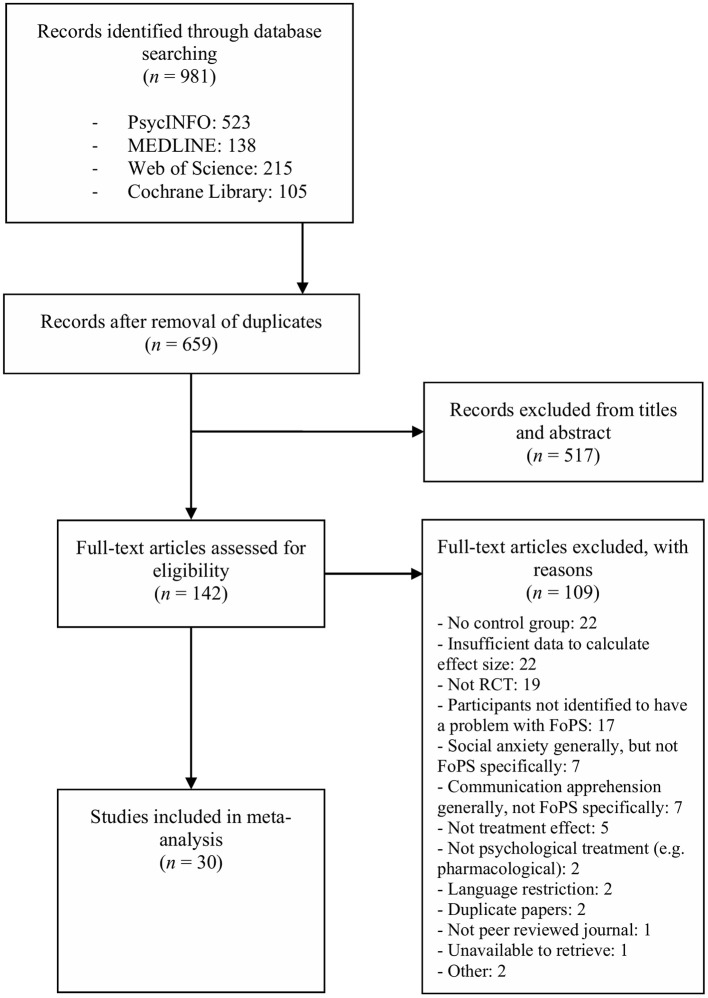
PRISMA flow diagram of the study selection process.

The systematic searches in PsycINFO, MEDLINE, Web of Science, and the Cochrane Library resulted in a total of 981 citations. After removal of duplicates, 659 citations remained. The screening of the titles and abstracts excluded 517 studies. The disagreement between the two independent reviewers appeared 21 times across the 659 citations, yielding an excellent Cohen's kappa of 0.91. A total of 142 full-text articles were retrieved and assessed for eligibility, wherein 109 articles did not meet our inclusion criteria and were consequently excluded. Concerning inclusion of full-text articles, the disagreement between the two independent reviewers appeared two times across 142 articles, yielding an excellent Cohen's kappa of 0.96. The specific reasons for exclusion are presented in Figure 1, which portrays the selection and inclusion process. In the exclusion category referred to as “Other,” two studies were excluded. The first study (Calff and MacLean, 1970) was excluded due to its faulty experimental design and inappropriate statistical analysis, as highlighted by Blanchard (1971). The second study (Straatmeyer and Watkins, 1974) was unavailable through retrieval processes and interlibrary loan. Finally, 33 studies met the inclusion criteria, amongst which three studies were separately published follow-up studies, yielding a total of 30 unique studies to be included in the meta-analysis. For FoPS, the 30 studies included 62 psychological interventions that were compared to a control group. For social anxiety, the 30 mentioned studies included 32 interventions that were compared to a control group.

### Study Characteristics

A total of 1,355 participants were included in the 30 RCTs included in this meta-analysis, where 869 belonged to the psychological intervention groups and 486 participants resided within the control conditions. Participants were identified through a diagnosis of social anxiety in four studies. In the remaining 26 studies, participants were identified through either a cut-off value on an instrument measuring FoPS (*N* = 14); through elevated scores on an instrument measuring FoPS (e.g., scoring one standard deviation above the mean *N* = 6); or through self-identification of FoPS as an impairing problem (*N* = 6). The target age group for provision of intervention was adults (individuals above 18 years) in 29 of the 30 studies. One study had adolescents as the target group. A total of 24 out of 30 studies reported gender. We calculated the percentage of females across these 24 studies to be 46.9%. Only three studies reported the ethnicity of the participants included. We calculated the percentage of ethnicities other than Caucasian across these three studies to be 34.5%. In all, 27 studies reported means and standard deviations, whereas the last three studies reported other statistical information (e.g., *F, p*, and *t* values).

Several studies had more than one intervention group. Of the 62 conditions across the 30 studies, 50 were based on cognitive or behavioral interventions, whereas 12 employed other types of interventions. Within the cognitive or behavioral interventions, the majority comprised variations of desensitization therapies (nine interventions), relaxation therapies (seven interventions), and cognitive-behavioral therapy (CBT; six interventions). Amongst the 12 non-cognitive or behavioral interventions, visualization therapy based on psychosynthesis (four interventions) and EMDR (four interventions) represented the majority. The remainder of intervention conditions as well as a full overview of all interventions within each study are provided in Table 1.

**Table 1:** Selected characteristics of the included randomized controlled trials assessing the effects of psychological treatments on fear of public speaking and generalized social anxiety.

**Study**	**Pop**.	**Conditions**	***N***	***N* ses**.	**Exposure type**	**Format**	**Mode delivery**	**Framework**	**Instr. FoPS**	**Instr. social anxiety**	**FU**
Anderson et al., 2013	D	VRET	30	8	VR	Individual	FTF	C/B	PRCS	BFNE	72^f^
		EGT	39	8	*In vivo*	Group	FTF	C/B	Speech length^b^		
		WL	28						Speech length-Peak anxiety^b^		
Ayres, 1995	ND	Vis.	20	1	Imaginary	Individual	FTF	PD	PRCA^*^	None	None
		Active vis.	20	1	Imaginary	Individual	FTF	PD	BASA^b^		
		Active vis. +	20	1	Imaginary	Individual	FTF	PD			
		vis.									
		WL	20								
		Placebo	20								
Ayres et al., 1994	ND	Performance	20	1	Imaginary	Individual	FTF	PD	PRCA^*^	None	None
		Vis.							BASA^b^		
		Placebo	19								
		WL	20	1							
Botella et al., 2010	D	SA-CBT	30	N/A	Video	Self-help	Int. delivered	C/B	FPSQ	BFNE	12
		CBT	22	16	Video	Individual	FTF	C/B	SSPS-P	SAD	
		WL	25						SSPS-N		
									IST-P^b^		
									IST-T^b^		
Cunningham et al., 2006	ND	TLM	17	3.3^c^	None	Individual	Telephone	Other	PRCS	None	None
		WL	19						SRP-F		
									SRP-A		
Deffenbacher et al., 1980	ND	Hom. AMT	13	6	Imaginary	Group	FTF	C/B	PRCS	None	12^f^
		Het. AMT	12	6	Imaginary	Group	FTF	C/B	TAS		
		NT	16								
Foley and Spates, 1995	ND	EMDR	9	NR	Imaginary	Individual	FTF	EMDR	BASA^b^	None	NR
		EMDR	9	NR	Imaginary	Individual	FTF	EMDR	HR-R		
		w/sound									
		EMDR	9	NR	Imaginary	Individual	FTF	EMDR			
		w/resting eyes									
		NT	9						^a^		
Fremouw and Harmatz, 1975	ND	Latent helpers	10	10	*In vivo*	Group	FTF	C/B	GRSA	SAD	3
		Helpers	10	10	*In vivo*	Group	FTF	C/B	PRCS	FNE	
		Helpees	10	5	*In vivo*	Group	FTF	C/B	AD		
		WL							TBCL^b^		
									OA		
Fremouw and Zitter, 1978	ND	Skills training	12	5	Imaginary	Group	FTF	C/B	PRCS	SAD	2
		Cogn.	12	5	*In vivo*	Group	FTF	C/B	AD		
		relaxation							BASA^b^		
		Placebo	11						OA^b^		
		WL	11						ToS^b^		
Gallego et al., 2011	D	SA-CBT	13	N/A	Video	Self-help	Int. delivered	C/B	PRCS	BFNE	None
		WL	11						SSPS-P	SAD	
									SSPS-N		
									IST-P^b^		
									IST-T^b^		
									IST-O^b^		
Gatchel and Proctor, 1976	ND	Biofeedback	18	3	Imaginary	Individual	FTF	C/B	USRA	None	1
		Placebo	18						TBCL^b^		
									HR^a^		
Goldfried and Trier, 1974	ND	Self-control	10	5	Imaginary	Group	FTF	C/B	TBCL^b^	SAD	1.5
		relaxation							Silence^b^	FNE	
		Standard	9	5	Imaginary	Group	FTF	C/B	AD		
		Relaxation							SAS		
		Placebo	8						PRCS		
									S-R		
									Inventory		
									TAS		
Gross and Fremouw, 1982	ND	Cog.	23	4	NR	NR	FTF	C/B	SAS	FNE	None
		Restructuring							PRCS	SAD	
		Progressive	26	4	NR	NR	FTF	C/B	HR^a^		
		relaxation							SCL^a^		
		WL	10						TBCL^b^		
									OA^b^		
Harris et al., 2002	ND	VRT	8	4	VR	Individual	FTF	C/B	PRCS	LSAS	None
		WL	6						STAI		
									ATPS		
									HR^a^		
Hayes and Marshall, 1984	ND	SIT	14	8	Imaginary	Group	FTF	C/B	SUDS	SAD	6
		SIT + Flooding	14	8	Mix	Group	FTF	C/B	FT	FNE	
		SIT + flooding	14	8	Mix	Group	FTF	C/B			
		+ skills									
		training									
		Placebo	14								
Hekmat et al., 1985	ND	Instructional	10	3	Imaginary	Individual	FTF	C/B	S-R	None	1
		desem.							Inventory		
		Placebo	10						ACL		
		WL	10						PRCS		
									TBCL^b^		
Homer et al., 2016	ND	EMDR	17	1	Imaginary	Individual	FTF	C/B	PRCS	None	None
		Placebo	19	1	Imaginary	Individual	FTF	C/B	SA		
Johnson et al., 1971	ND	Group desen.	8	9	Imaginary	Group	FTF	C/B	PRCS	None	None
		Speech	8	9	*In vivo*	Group	FTF	C/B			
		practice									
		NT	10								
Karst and Trexler, 1970	ND	Fixed-role	8	3	None	Group	FTF	C/B	PRCS	SFS	N/A^d^
		therapy									
		RET	8	3	None	Group	FTF	C/B	AS	SFS	
		NT	6								
Kirsch and Henry, 1977	ND	Systematic desen.	11	5	Imaginary	Individual	FTF	C/B	AD PRCS	None	None
		Operant desen.	11	5	Imaginary	Individual	FTF	C/B	TBCL^b^		
		Placebo	11								
Marshall et al., 1976	ND	TA desensitization	9	5	Imaginary	Group	FTF	C/B	TBCL^b^ FT	None	None
		SA desensitization	6	N/A	Imaginary	Self-help	SHM	C/B	SUDS		
		SA desen. + check	9	5	Imaginary	Self-help	SHM	C/B			
		SA desen. + placebo	9	5	Imaginary	Group	FTF	C/B			
		Placebo	9								
		NT	9								
McNally et al., 2013	ND	ABM	7	4	None	Individual	Computerized	C/B	PRCS	LSAS	None
		I-ABM	7	4	None	Individual	Computerized	C/B	USRA-B		
		Placebo	7						USRA-A		
									SPRS^b^		
									HR-B^a^		
									HR-A^a^		
									SysB^a^		
									SysA^a^		
									DiaB^a^		
									DiaA^a^		
Meichenbaum et al., 1971	ND	Desensitization	11	8	*In vivo*	Group	FTF	C/B	PRCS	SAD^e^	3
		Insight	11	8	Imaginary	Group	FTF	Gestalt	ACL	FNE^e^	
		Des. + insight	10	8	*In vivo*	Group	FTF	Gestalt	AD		
		Placebo	10						TBCL^b^		
		WL	7						Word count^b^		
									Silence^b^		
									Ah-statements^b^		
Newman et al., 1994	D	Exposure only	16	8	Mix	Group	FTF	C/B	SAS	SPAI	None
		WL	17						TAS	SAD	
									PRCS	FNE	
									GST^b^		
Osberg, 1981	ND	Applied relaxation	15	6	Mix	Group	FTF	C/B	PRCS S-R	SAD	1
		Standard relaxation	13	6	Imaginary	Group	FTF	C/B	Inventory STAI		
		Speech practice	15	6	Mix	Group	FTF	C/B	TBCL^b^ GR^b^		
		WL	14								
Schoenberger et al., 1997		CBT	17	5	*In vivo*	Group	FTF	C/B	AE	FNE	None
		CBT	15	5	*In vivo*	Group	FTF	C/B	PRCS		
		w/hypnosis							SUDS		
		WL	10						TBCL^b^		
									PR^a^		
Schwartz and Kaloupek, 1987	ND	Exercise and exposure	13	4	Imaginary	Individual	FTF	C/B	PRCS S-R	None	None
		Exposure alone	13	4	Imaginary	Individual	FTF	C/B	Inventory TBCL^b^		
		Exercise alone	13	4	None	Individual	FTF	Other	Silence^b^		
		Placebo	13	4					Prepatory HR^a^		
									Speech HR^a^		
									Prepatory SCL^a^		
									Speech SCL^a^		
									Prepatory SCR^a^		
									Speech SCR^a^		
Spector et al., 1993	ND	CCR-W	9	6	None	NR	FTF	C/B	PRCS	FNE	2
		CCR-A	8	6	None	NR	FTF	C/B	S-R		
		Placebo	7						Inventory		
		WL	8						BRS^b^		
Trussell, 1978	ND	GBR/F	15	8	*In vivo*	Group	FTF	C/B	PRCS	None	1.5
		GBR/F + systematic des.	15	8	Imaginary	Group	FTF	C/B	SAS TAS		
		NT	13						BASA^b^		
Wallach et al., 2009	ND	VRCBT	28	12	VR	Individual	FTF	C/B	SSPS-P	FNE	12^f^
		CBT	30	12	Imaginary	Individual	FTF	C/B	SSPS-N	LSAS-Fear	
		WL	30						OR^b^	LSAS-Avoid.	
									SRA^b^		

*ABM, Attention Bias Modification; ACL, Affect adjective Checklist; AD, Anxiety Differential; AE, Anxiety Expectancy; AS, Anxiety Scale with 10 points similar to Fear Thermometer; ATPS, Attitudes Toward Public Speaking; BASA, Behavioral Assessment of Speech Anxiety; BFNE, Brief Fear of Negative Evaluation; BRS, Behavior Rating System; CBT, Cognitive Behavioral Therapy; CCR-W, Cue-Controlled Relaxation - Word Cue; CCR-A, Cue-Controlled Relaxation – Aroma Cue; C/B, Intervention belonging to the Cognitive or Behavioral family; D, Diagnostic; DiaA, Diastolic blood pressure after speaking; DiaB, Diastolic blood pressure before speaking; EGT, Exposure Group Therapy; EMDR, Eye Movement Desensitization and Reprocessing; FNE, Fear of Negative Evaluation; FT, Fear Thermometer; FTF, Face to face; FU, Follow-Up in months; GBR/F, Graduated Behavior Rehearsal/Feedback; GR, Global Rating of anxiety; GRSA, General Rating of Speech Anxiety; GST, Graded Speech Task; Het. AMT, Heterogeneous Anxiety Management Training; Hom. AMT, Homogenous Anxiety Management Training; HR, Heart Rate; HR-A, Heart Rate After speaking; HR-B, Heart-Rate Before speaking; HR-R, Heart Rate Reactivity; Instr., instrument; Int. delivered, Internet-delivered therapy; IST-P, Impromptu Speech Task – Patient reported; IST-T, Impromptu Speech Task – Therapist reported; IST-O, Impromptu Speech Task—Observer reported; I-ABM, Inverse Attention Bias Modification; LSAS, Liebowitz Social Anxiety Scale; Mix, Mixture of in vivo and imaginary exposure; N/A, Not available; ND, Non-diagnostic; NR, Not Reported; NT, No Treatment control group; OA, Overall Anxiety; OR, Observer Rating of anxiety during behavioral task; PD, Psychodynamic; Pop., Population; PR, Pulse Rate; PRCA, Personal Report of Communication Apprehension; PRCS, Personal Report of Confidence as Speaker; RET, Rational-Emotive Therapy; SA, State Anxiety on a visual analog scale; SAD, Social Avoidance and Distress scale; SAS, State Anxiety Scale; SCL, Skin Conductance Level; SCR, Skin Conductance Response; SFS, Social Fear Scale; SHM, Self-Help Material; STAI, State-Trait Anxiety Inventory; SPAI, Social Phobia and Anxiety Inventory; SPRS, Social Performance Rating Scale; SUDS, Subjective Units of Discomfort Scale; SysA, Systolic blood pressure after speaking; SysB, Systolic blood pressure before speaking; SA-CBT, Self-administered CBT; SRA, Self-Rated measure of Anxiety during behavioral task; SRP-A, Self-Rated Performance – Anxiety; SRP-F, Self-Rated Performance—Fear; S-R Inventory, S-R Inventory of speech anxiousness; TA, Therapist-administered; TAS, Trait Anxiety Scale; TBCL, Timed Behavior Checklist; TLM, The Lefkoe Method; ToS, Time of Silence; USRA, Unspecified Self-Report measure of Anxiety; USRA-A, Unspecified Self-Report measure of Anxiety After speaking; USRA-B, Unspecified Self-Report measure of Anxiety Before speaking; Vis., Visualization; VR, Virtual Reality; VRCBT, Virtual Reality Cognitive Behavioral Therapy; VRET, Virtual Reality Exposure Therapy; WL, Waitlist control*.

* *The PRCA was only used when the study reported the results based on its public speaking subscale alone*.

a *Physiological outcome measures*.

b *Behavioral outcome measure assessing overt signs of anxiety*.

c *Study authors only provided the mean number of sessions for all participants, with a range of 2–5 sessions*.

d *The follow-up results of this study were described qualitatively. No quantitative data were available here*.

e *The authors included these measures but did not report their outcomes*.

f *Reported in separate follow-up paper*.

Most of the included interventions (*N* = 28) were based on a group format. Furthermore, 24 of the 62 interventions employed an individual treatment format, and five studies used self-help materials. The remaining five interventions did not report type of format used. The number of treatment sessions ranged from 1 to 16, where the majority of the interventions had eight or fewer sessions (*N* = 49). A total of seven interventions had more than eight sessions. The remaining six interventions did not report the number of sessions employed.

The majority of the included interventions were non-technological (*N* = 55), nearly all of which consisted of face-to-face individual or group interventions, except for one intervention which was delivered via telephone. The remaining seven interventions were categorized as technology-delivered interventions, of which three encompassed virtual reality-based interventions, two represented Internet-based cognitive self-help interventions, and two utilized a computer application for attention bias modification.

Concerning control groups, 16 of the 30 studies included a waiting-list or no-treatment control group only, seven studies included a placebo group only, and the remaining seven studies included both a placebo and a waiting list (or no-treatment) group. Six of the 14 placebo groups were categorized as attention placebo (e.g., discussion groups), six placebo conditions were described by the study authors as credible replacements of the interventions (sham), and the last two placebo groups involved listening to tapes on communication unrelated to public speaking.

In the present meta-analysis, half of the included studies (*N* = 15) included a follow-up measure. The mean follow-up length was 9.28 months. However, due to an extreme outlier (follow-up of 72 months; 6 years), we also calculated the median follow-up length which was 2.5 months. Removing this outlier, the mean follow-up length was 4.46 months. Furthermore, 26 of the included studies were conducted in the USA or Canada, three in Europe and one in Asia. Only one out of the 30 included studies had included an intention-to-treat analysis, while the remaining 29 studies were based on completer data. No study explicitly reported data on researcher allegiance. Three out of 30 studies reported therapist effects, where one study reported holding therapist effects constant across groups, while the two other studies revealed insignificant findings for these effects. Table 1 provides a complete overview of study characteristics.

### Risk of Bias

**Figure 2:**
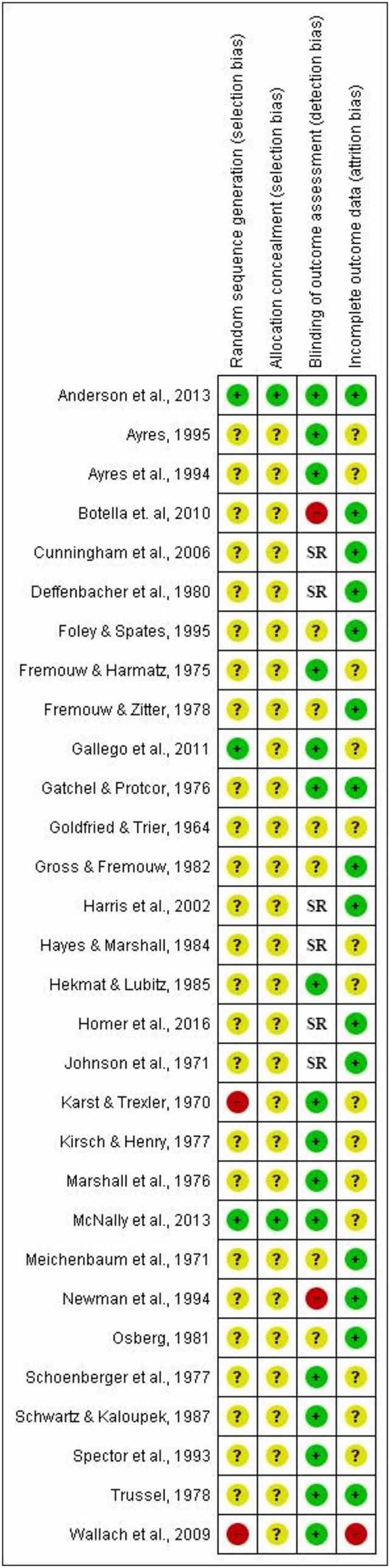
Estimated risk of bias of the included studies. SR, Self-report.

Disagreement between the two independent reviewers occurred three times in the coding of risk of bias, yielding an excellent Cohen's kappa of 0.93. In the domain regarding masking of outcome assessors, 16 studies were judged to have a low risk of bias, meaning that the outcome assessors had no knowledge of the allocated intervention by being blinded. Of the remaining studies, six studies were found to have an unclear risk of bias, while two studies were judged to have a high risk of bias. The last six studies in this domain were solely based on self-report measures. Regarding attrition bias or the domain of dealing with incomplete data, 15 studies were found to have a low risk of bias meaning that the studies had outcome data for all or nearly all participants. Furthermore, 14 studies were judged to have an unclear risk of bias, whereas one study was found to have a high risk of bias. Only one of the 30 studies included had an intention-to-treat analysis, while the remaining 29 studies were based on completer data. Concerning the domain of adequate generation of allocation sequence, it was found that 25 studies had an unclear risk of bias, implying that no information was provided on how the sequence generation of randomization was conducted. Furthermore, three studies were judged to have a low risk of bias in this domain, whereas two studies were found to have a high risk of bias, the latter portraying that the sequence generation was done in a manner that was not truly random as exemplified by the Cochrane risk of bias tool. For the concealment of allocation to conditions domain, we found 28 studies to have an unclear risk of bias where no information was presented on whether the allocation to conditions were concealed. The remaining two studies demonstrated a low risk of bias. Risk of bias of the included studies is depicted in Figure 2.

### Synthesized Findings

#### Post-treatment Effects of Psychological Interventions Compared With Control Groups on FoPS

**Figure 3:**
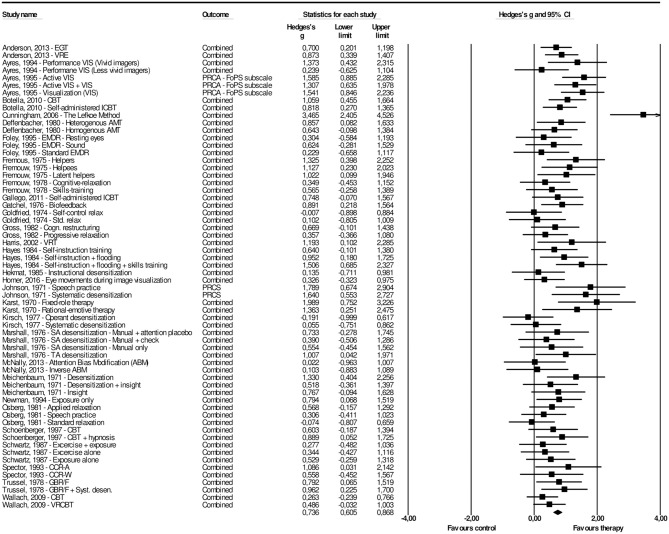
A forest plot of the standardized effect sizes of psychological treatments for fear of public speaking compared with control conditions.

Table 1 provides a summary of the 30 studies that were included in the present meta-analysis, including 62 interventions across 30 studies. The overall effect of psychological interventions for FoPS was *g* = 0.74 (95% CI: 0.61–0.87), with a low to moderate amount of heterogeneity (*I*^2^ = 40.85, 95% CI: 19.66–56.45) that was found to be significant (*Q* = 103.12, *p* = 0.001). This corresponds to an NNT of 2.50. For each study, the effect size with its associated 95% confidence interval is presented in Figure 3. There was one potential outlier that did not overlap with the 95% CI of the pooled effect size (Cunningham et al., 2006). Removing this outlier resulted in an overall effect size of *g* = 0.70 (95% CI: 0.59–0.82), with low heterogeneity (*I*^2^ = 22.24, 95% CI: −7.70–58.52) that was non-significant (*Q* = 77.16, *p* = 0.067).

In 22 of the 30 studies reported above, two or more psychological interventions were compared to the same control group. A potential consequence of such multiple comparisons might be an artificial reduction of heterogeneity, which in turn can affect the overall effect size. In order to take this into account, we followed the procedure of Cuijpers et al. (2014) and conducted an analysis that included only one effect size per study. We examined this by first including the largest effect size from the studies, yielding a result of *g* = 0.91 (95% CI: 0.70–1.12), with a moderate amount of heterogeneity (*I*^2^ = 50.32, 95% CI: 27.92–67.39) that was significant (*Q* = 58.37, *p* = 0.001). The analysis including only the smallest effect size resulted in an overall effect size of *g* = 0.63 (95% CI: 0.43–0.83), with a moderate amount of heterogeneity (*I*^2^ = 50.16, 95% CI: 27.57–67.30) that was significant (*Q* = 58.19, *p* = 0.001).

A visual inspection of the funnel plot, in addition to Duval and Tweedie's trim and fill procedure revealed some signs of possible publication bias for the effect of psychological interventions on FoPS at post-test. The funnel plot revealed three potential missing studies and is presented in Supplementary Figure 1. Duval and Tweedie's procedures informed that three studies were missing to the right of the mean. Therefore, the calculated effect size after the adjustment of publication bias was slightly higher, *g* = 0.76 (95% CI: 0.66–0.86) for psychological interventions on FoPS at post-treatment. Finally, the Orwin's fail safe *N* to quantify the amount of studies with zero effect (*g* = 0.00) that would be needed to bring the observed effect size down to the chosen “trivial” value (*g*) of 0.20 (a finding of trivial clinical importance) showed this number to be 162.

We also examined whether there were differences in the effect size of psychological interventions for FoPS between studies that included a diagnostic sample (i.e., participants were formally diagnosed with social anxiety disorder) and studies that included subclinical samples (e.g., participants scored above a cut-off and did not fulfill formal diagnostic criteria). No significant differential (*p* = 0.579) effect was found.

#### Post-treatment Effects of Psychological Interventions Compared With Control Groups on Generalized Social Anxiety

**Figure 4:**
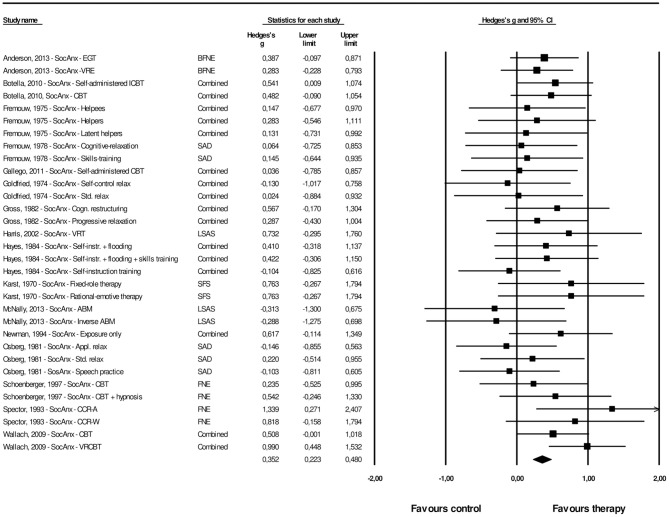
A forest plot of the standardized effect sizes of psychological treatments aimed at FoPS for generalized social anxiety compared with control conditions.

A total of 32 interventions across 16 studies provided sufficient data concerning the effects on generalized social anxiety. The overall effect size for psychological interventions aimed at FoPS on generalized social anxiety was *g* = 0.35 (95% CI: 0.22–0.48). Heterogeneity was zero and non-significant (*Q* = 25.60, *p* = 0.740). This corresponds to an NNT of 5.11. The effect sizes along with their associated 95% confidence intervals are presented in Figure 4.

The procedures by Duval and Tweedie and inspection of the funnel plot was conducted for the effect size of psychological interventions aimed at FoPS on generalized social anxiety. The funnel plot revealed some signs of possible publication bias and can be found in Supplementary Figure 2. Duval and Tweedie's procedures revealed that three studies were missing to the right of the mean. The adjusted effect size for psychological interventions aimed at FoPS for generalized social anxiety was thus *g* = 0.39, 95% CI: 0.27–0.52). Orwin's fail safe *N* quantifying the amount of studies with zero effect (*g* = 0.00) that would be needed to bring the observed effect size down to the chosen “trivial” value (*g*) of 0.20 (a finding of trivial clinical importance) was found to be 25.

#### Long-Term Effects of Psychological Interventions on FoPS

**Figure 5:**
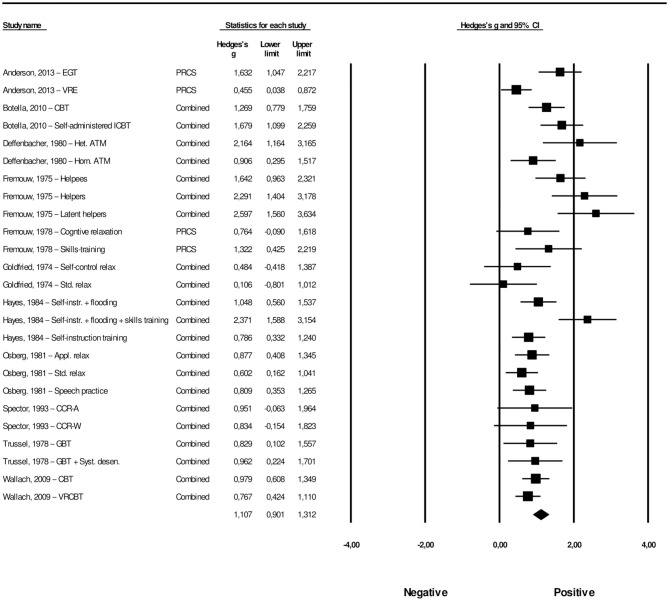
A forest plot of the standardized effect sizes of psychological treatments aimed at fear of public speaking at follow-up.

Our meta-analysis of the long-term effects of psychological interventions on FoPS yielded an overall effect of *g* = 1.11 (95% CI: 0.90–1.31), with a moderate amount of heterogeneity (*I*^2^ = 64.76, 95% CI: 46.12–76.95; *Q* = 68,10, *p* < 0.001). This corresponds to an NNT of 1.76. For each study, the effect size along with its associated 95% confidence intervals can be found in Figure 5. Since the vast majority of the included studies used a waiting list control group, participants in these conditions had received treatment at follow-up, meaning only within-group data was available for these studies. A total of 11 studies reported data that would allow for long-term effect comparisons. Only eight conditions across four of these studies had compared the intervention group with a control group at follow-up (between-group comparisons). The remaining 17 conditions across seven studies did not have a control group at this time point. We therefore conducted within-group effect size calculations for these 17 conditions, combining them with the between-group calculations of the eight other conditions. In these calculations, we coded whether the effect size was based on a between-group or within-group comparison as a moderator, to be used in a meta-regression analysis reported later in this paper.

Visual inspection of the funnel plot, in addition to Duval and Tweedie's trim and fill procedure revealed no potential signs of publication bias for the effect size concerning the long-term effects of psychological interventions on FoPS. The funnel plot is presented in Supplementary Figure 3. Orwin's fail safe *N* quantifying the amount of studies with zero effect (*g* = 0.00) that would be needed to bring the observed effect size down to the chosen “trivial” value (*g*) of 0.20 (a finding of trivial clinical importance) showed this number to be 228.

#### Long-Term Effects of Psychological Interventions on Generalized Social Anxiety

**Figure 6:**
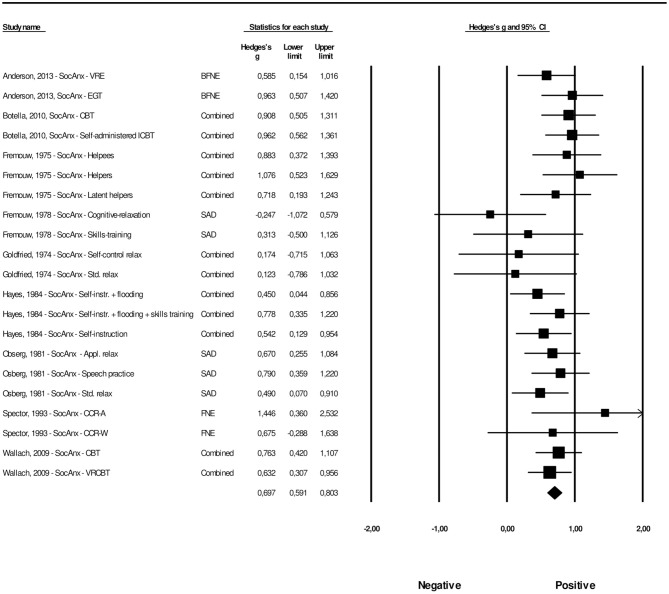
A forest plot of the standardized effect sizes of psychological treatments aimed at fear of public speaking on generalized social anxiety at follow-up.

The meta-analysis of the long-term effects on generalized social anxiety resulted in an overall effect size of *g* = 0.70 (95% CI: 0.59–0.80), where heterogeneity was non-significant (*Q* = 20,72, *p* = 0.414). This corresponds to an NNT of 2.64. The effect sizes along with their associated 95% confidence intervals can be found in Figure 6. For generalized social anxiety, a total of nine studies provided data for long-term effects comparisons. Only six conditions across three studies had between-group data available at follow-up, comparing the intervention and control groups. We used within-group effect size calculations for the remaining 15 conditions across six studies. Once again, we registered whether effect size calculation was based on a between-group or a within-group comparison as a moderator.

The procedures by Duval and Tweedie and inspection of the funnel plot were conducted for the effect size concerning the long-term effects of psychological interventions aimed at FoPS on generalized social anxiety. The funnel plot revealed a relatively symmetrical distribution; still there were some signs of potential publication bias. The funnel plot can be found in Supplementary Figure 4. Duval and Tweedie's procedures revealed two potential missing studies to the right of the mean. The effect size concerning the long-term effects of psychological interventions aimed at FoPS on generalized social anxiety was thus adjusted to *g* = 0.72 (95% CI: 0.62–0.82). Orwin's fail safe *N* quantifying the amount of studies with zero effect (*g* = 0.00) that would be needed to bring the observed effect size down to the chosen “trivial” value (*g*) of 0.20 (a finding of trivial clinical importance) was found to be 126.

### Meta-Regression Results

A series of planned meta-regression analyses were conducted with moderator variables chosen a priori to address our aims of investigating the differences in effect size for different modes of delivery, theoretical frameworks, type of control groups, and types of outcome measures. A limited number of moderators were analyzed in order to maintain a reasonable ratio between of single effect sizes/moderators.

#### Meta-Regression Analysis for the Effects of Psychological Interventions on FoPS at Post-treatment

First, we conducted a meta-regression analysis with the effects of psychological interventions on FoPS at post-treatment. We included the following variables in this analysis: (1) theoretical framework (cognitive or behavioral vs. other theoretical models); (2) type of control group (placebo vs. no-treatment or waiting list); (3) percentage of measures other than self-report; and (4) technology-delivered vs. non-technological interventions. Theoretical framework was a significant predictor of the effect size on FoPS at post-treatment (*p* = 0.016), favoring “other” theoretical models (insight therapy, visualization therapy, The Lefkoe Method, and EMDR) over cognitive or behavioral models. However, removing the extreme outlier (The Lefkoe Method; Cunningham et al., 2006) that did not overlap with the 95% CI of the pooled effect size, we found no significant differences between “other” theoretical models compared to cognitive or behavioral models (*p* = 0.104). We examined whether this finding could be explained by the studies with cognitive or behavioral interventions more often including physiological or behavioral outcome measures. In accordance with this, we compared the two groups only including self-report measures. Still, no significant difference in effect size between cognitive or behavioral interventions and “other” intervention (*p* = 0.821) was found.

The type of control group used was found to be a significant predictor of the effect size, where waiting-list or no-treatment control groups had a more favorable effect compared to placebo groups (*p* = 0.012), as expected. Following up on this with a subgroup analysis, we found that the effect of psychological interventions compared to placebo groups was moderate to large (*g* = 0.65, 95% CI: 0.46–0.84), whereas the same effect compared to waiting-list or no-treatment control group was large (*g* = 0.82, 95% CI: 0.63–1.01).

Returning to the meta-regression, the percentage of outcome measures other than self-report was found to be a significant predictor of the effect size (*p* < 0.000), with studies that had a higher percentage of other outcome measure types than self-report (i.e., physiological or behavioral measures) yielding lower effect sizes.

Finally, we found no significant differences between face-to-face and technology-delivered interventions (i.e., Interned-delivered therapies, virtual reality exposure therapies and computerized interventions), *p* = 0.814. The meta-regression model explained 56% (*R*^2^ = 0.56) of the observed heterogeneity. The Goodness of fit test showing whether still unexplained variance was significantly different from zero was non-significant (*p* = 0.065). Meta-regression results with standard regression coefficients, confidence intervals, and associated *p*-value are presented in Table 2.

**Table 2:** Multivariate meta-regression analyses of the four overall effect sizes calculated in this study, with standard regression coefficients and their associated 95% CI and *p*-value are provided.

			**FoPS at post-treatment**			**FoPS at follow-up**		
	**Category (*k*)**	***g***	**Coeff**.	**95% CI**	***p***	**Coeff**.	**95% CI**	***p***
Theoretical framework	C/B (50) Other (12)	0.673 0.693	Ref.0.369	0.068 to 0.671	0.016			
Type of control group	Placebo (33) WL/NT (29)	0.649 0.822	Ref.0.315	0.068 to 0.563	0.012			
Percentage of measures other than self-report (continuous)			−0.010	−0.015 to −0.005	0.000			
Technology-delivered vs. non-technological interventions	Non-tech. (55) Tech. (7)	0.753 0.658	Ref.0.043	−0.315 to 0.401	0.814			
Follow-up length (continuous)						−0.003	−0.015 to 0.007	0.509
Type of effect size at follow-up	Between (8) Within (17)	0.791 1.225				Ref. 0.479	−0.028 to 0.988	0.064
Intercept			0.862	0.612 to 1.106	0.000	0.792	0.369 to 1.214	0.000

*Between, Between group comparison of intervention vs. control group; CI, Confidence Interval of regression coefficient; Coeff., regression coefficient; C/B, Cognitive or Behavioral; FoPS, Fear of public speaking; g = Hedges' g; k = number of interventions; Non-tech, Non-technological intervention; Ref., Reference group; Tech. Technological intervention; Within, Within group comparison between based on pre-intervention vs. follow-up assessment; WL/NT, Waiting list control group or No-Treatment control group*.

#### Meta-Regression Analysis for the Long-Term Effects of Psychological Interventions on FoPS

For the second meta-regression concerning the long-term effects of psychological interventions for FoPS, we had pre-selected the following moderator variables; (1) time from post-treatment to follow-up (follow-up length); and (2) whether the effect size at follow-up was calculated within-groups (comparing pre-treatment scores to follow-up scores) or between-groups (comparing intervention group to a control group). Once again, we did not investigate other moderator variables to maintain a reasonable ratio between effect sizes and the number of moderators. As seen in Table 2, the results from this analysis show that follow-up length was not a significant predictor of the effect size (*p* = 0.509).

The other moderator variable in this analysis (type of effect size; within or between) was also non-significant (*p* = 0.064). This indicates that no difference was found between the effects calculated from between-group comparisons and the effects calculated from within-groups comparisons. As moderators in this model were non-significant, we could not account for any of the observed heterogeneity shown by a significant Goodness of fit test (*p* < 0.001), which indicates that there still remained unexplained variance after accounting for the two moderators.

#### Meta-Regression Analysis for Generalized Social Anxiety

Since meta-regression analyses were conducted only in cases of significant heterogeneity, no such analyses were conducted for the effects of psychological interventions aimed at FoPS on generalized social anxiety at post-treatment or for the long-term effects on generalized social anxiety at follow-up.

### Changes in Effect After Treatment Termination

Two meta-analyses were conducted to investigate the changes in effect after treatment termination from post-treatment to follow-up, also referred to as the “sleeper effect” (e.g., Flückiger et al., 2015).

#### Changes in Effect After Treatment Termination for Psychological Interventions on FoPS

**Figure 7:**
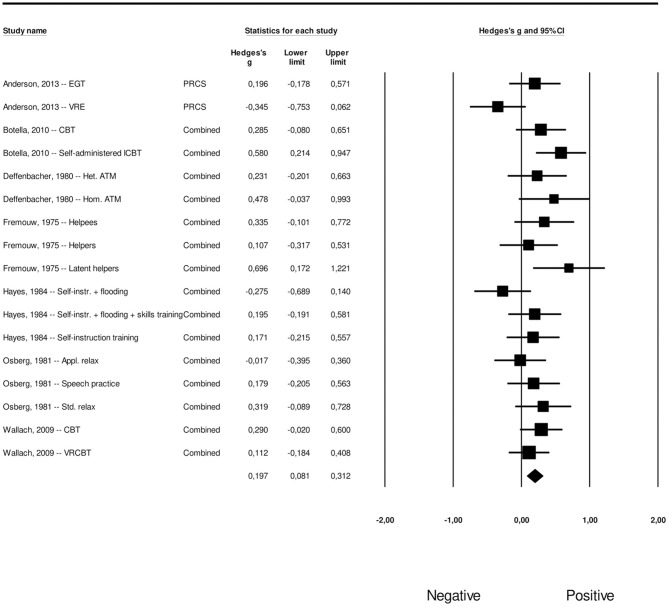
A forest plot of the change in efficacy from post-treatment to follow-up of psychological treatments aimed at fear of public speaking.

A meta-analysis was conducted to examine changes in efficacy of psychological interventions for FoPS over time after treatment termination (post-treatment to follow-up). The results revealed an overall effect size of *g* = 0.20 (95% CI: 0.081–0.312), where heterogeneity was non-significant (*Q* = 23.69, *p* = 0.096). This corresponds to an NNT of 8.89. The effect sizes and their associated 95% confidence intervals can be found in Figure 7.

Visual inspection of the funnel plot, in addition to Duval and Tweedie's trim and fill procedure revealed some signs of publication bias for the effect size concerning changes in efficacy of psychological interventions on FoPS from post-treatment to follow-up. The funnel plot revealed four potential missing studies and is presented in Supplementary Figure 5. Duval and Tweedie's trim and fill procedure revealed four potential missing studies to the left of the mean. The effect size concerning changes in efficacy of psychological interventions on FoPS from post-treatment to follow-up was adjusted to *g* = 0.12 (95% CI: 0.04–0.21) when applying the trim and fill procedure.

#### Changes in Effect After Treatment Termination for Psychological Interventions On Generalized Social Anxiety

**Figure 8:**
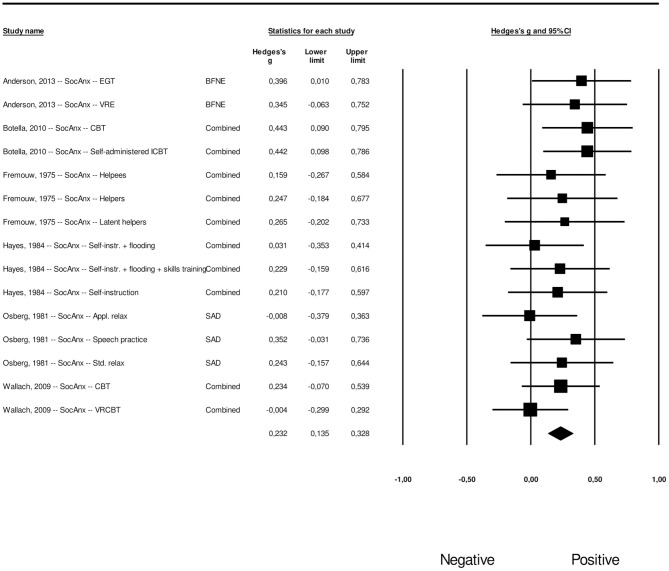
A forest plot of the change in efficacy from post-treatment to follow-up of psychological treatments aimed at fear of public speaking on generalized social anxiety.

We conducted a meta-analysis to assess the changes in efficacy of psychological interventions for generalized social anxiety outcomes from post-treatment to follow-up. The results yielded an average effect size of *g* = 0.23 (95% CI: 0.135–0.328), where heterogeneity was non-significant (*Q* = 9.44, *p* = 0.802). This corresponds to an NNT of 7.74. The effect sizes along with their associated 95% confidence intervals are presented in Figure 8.

The funnel plot revealed four potential missing studies and is presented in Supplementary Figure 6. Duval and Tweedie's trim and fill procedure revealed four potential missing studies to the left of the mean. The effect size concerning changes in efficacy of psychological interventions for generalized social anxiety from post-treatment to follow-up was *g* = 0.16 (95% CI: 0.08–0.25) after adjustment based on the trim and fill procedure.

### Secondary Outcome Measures

As specified in the objectives and the study protocol, when available, depression, satisfaction with treatment, treatment credibility, and outcome-expectancy outcome measures were coded with the additional aim to conduct meta-analyses on these secondary measures. However, there were not enough (< 5 conditions) studies providing data for these outcomes to warrant such analyses. There were two studies reporting outcomes on depression, two studies reported satisfaction with treatment, three studies reported treatment credibility, and three studies reported outcome expectancy. Among these numbers there were also some qualitative descriptions for some of these measures (e.g., satisfaction with treatment). We therefore did not conduct meta-analyses for any secondary outcomes.

## Discussion

The present meta-analysis is the first on FoPS in nearly three decades (Allen et al., 1989). Furthermore, it is the first meta-analysis to date to examine the efficacy of psychological interventions for FoPS exclusively based on RCTs, producing more reliable results. This is in contrast to the meta-analysis by Allen et al. (1989), which included uncontrolled and non-randomized studies. Our literature search identified 30 studies researching the effects of psychological interventions on FoPS, including *N* = 14 new RCTs since the last meta-analysis conducted by Allen et al. (1989).

### Summary of Main Findings

#### The Effects of Psychological Interventions on FoPS at Post-treatment

The first aim of the present meta-analysis was to assess the overall short-term effect of psychological interventions for FoPS when compared to a control group. We found moderate to large effects on FoPS (Hedges *g* = 0.74), corresponding to an NNT of 2.50, indicating that between two to three patients must be treated in order to generate one positive patient outcome. This finding seems robust as the FoPS interventions were compared to a relatively large pool of both active and passive control groups, where nearly half were from active placebo groups. When comparing the psychological interventions for FoPS at post-treatment to passive control groups only (e.g., waiting list), a large effect size was revealed. On the other hand, comparing psychological interventions for FoPS at post-treatment to an active control group only (e.g., attention placebo) resulted in a moderate to large effect size. This is an important conclusion, suggesting that psychological interventions are effective in reducing FoPS on a clinically significant level when compared to both passive waiting list groups and active placebo groups, indicating robustness.

Furthermore, we conducted a subgroup analysis to investigate whether there were differences in the effect of psychological interventions for FoPS between studies that used a diagnostic sample on the one hand and studies that used a subclinical sample on the other. No significant differences were found, suggesting that psychological interventions for FoPS are equally effective for individuals with a social anxiety diagnosis and individuals with subclinical symptoms.

#### The Long-Term Effects of Psychological Interventions on FoPS

The second aim of the present study was to investigate the long-term effects of the psychological interventions for FoPS. The psychological interventions were indeed effective at follow-up for FoPS, demonstrating a large effect size (Hedges *g* = 1.11). A meta-regression revealed that follow-up length was not associated with the effect size, suggesting that the effects of psychological interventions on FoPS are stable and persist over time. Although we based this calculation primarily on within-group comparisons and, where available, between-group comparisons, we did not find any indication of a difference between these two types of comparisons in our meta-regression, providing further confidence for these long-term results.

#### Technology-Delivered and Traditional Face-to-Face Interventions

The third aim of this review was to find out whether there is a difference in effect between technology-delivered interventions (i.e., Interned-delivered therapies, virtual reality exposure therapies and computerized interventions) and individual or group face to face or telephone interventions. For the effect size on FoPS at post-treatment, we did not find any difference in effect size between technology-delivered interventions vs. more traditional face-to-face and telephone interventions. This provides support for technology-delivered psychological interventions as effective in treating FoPS. An important practical implication of this finding is highlighted by the advantages related to dissemination and individualization of treatment, presenting clinicians with an opportunity to provide different types of treatments to different patients. A patient with a severe level of social anxiety who may not be willing to initiate face-to-face therapy, may thus benefit from an intervention with a less threatening mode of delivery, such as an Internet-delivered intervention. Our findings are in line with a recent meta-analysis not finding any difference between Internet-delivered therapies vs. traditional face-to-face therapies for depression and anxiety disorders, including social anxiety disorder (Andrews et al., 2018).

#### Cognitive-Behavioral Interventions and Other Types of Interventions

The meta-regression analysis concerning the effect of psychological interventions for FoPS at post-treatment was utilized to test our fourth aim of investigating whether there was a difference between cognitive or behavioral interventions vs. “other” interventions. The multivariate regression analysis favored the “other” group consisting of insight therapy, visualization, The Lefkoe Method, and EMDR. However, removing an extreme outlier of which the 95% CI did not overlap with the overall pooled effect size (Cunningham et al., 2006), showed that no such differences were observed between the two categories of interventions. The latter analysis thus provides no indication of differences between cognitive or behavioral interventions vs. other interventions of FoPS. We examined whether this finding could be related to the studies using cognitive or behavioral interventions more often involving behavioral and physiological measures. Taking this into account by comparing the two groups only including self-report measures, we once again found no difference in treatment effects between cognitive or behavioral interventions and other interventions in treating FoPS. We recommend clinicians and researchers to interpret this finding with caution as the “other” group consists of a highly heterogeneous collection of interventions. More studies are therefore required to draw conclusions about the effects of interventions that are not cognitive or behavioral. It is furthermore important to highlight that, although the cognitive and behavioral group was coded in accordance with a previous meta-analysis by Cuijpers et al. (2014), also this category includes some heterogeneity, implying that the average effect sizes should be interpreted with caution and examined by forthcoming research. Future research should also investigate whether specific interventions for FoPS provide different effects in different population of patients.

#### Self-Reported vs. Physiological and Behavioral Measures

Our fifth aim—to investigate if there is a difference in effect size between self-report measures as compared to other outcome measures—was examined with the meta-regression analysis concerning the effect of psychological interventions for FoPS at post-treatment. We found that the effect size for FoPS at post-treatment was inversely related to the percentage of instruments included in a study that was not based on self-report. In other words, the effect size got increasingly lower the higher the amount of physiological and behavioral measures. The causes of such differences are not clear. One possible explanation regards the different nature of measures. Whereas self-report measures assess the individual's perceived state of fear and anxiety, the behavioral or observational measures are primarily concerned with overt or visible signs of anxiety and how the individual performed in a public speaking situation. This view is shared by Lang (1968) who argues that anxiety is conceptually linked to three related, but different, response systems: the behavioral, the subjective, and the physiological. Thereby, this may reflect a difference between signs of anxiety that are visible and signs of anxiety that are covert. Also, behavioral measures are commonly rated by an observer or clinician. Thus, another possibility for the differences could be that the raters are more negatively biased or conservative, whereas the participants are positively biased. Furthermore, it cannot be ruled out that discrepancy in effects between different sources of information might reflect differences in sensitivity with regard to the instruments used, with some instruments being more sensitive than others. Nevertheless, our findings correspond with an earlier study (Heimberg et al., 1990), where self-report measures were found to produce greater reductions in social anxiety than behavioral and physiological measures. These differences highlight the importance of assessing different types of outcome measures in a meta-analysis, which is one of the major assets of the present meta-analysis. The aforementioned differences also suggest that future studies on FoPS should attempt to include different measures of anxiety, for instance in accordance with the three systems-model of fear proposed by Lang (1968). It would also be of interest to investigate whether different symptoms profiles within such systems have implications for daily functioning and treatment.

#### The Effects of Psychological Interventions Aimed at FoPS on Generalized Social Anxiety

The sixth aim of the present study was to examine whether psychological interventions aimed at FoPS have an effect on concurrent generalized social anxiety, and whether these effects would be maintained at follow-up. It was found that the effects on generalized social anxiety outcomes had a small to moderate (Hedges *g* = 0.35) effect. This corresponds to an NNT of 5.11, meaning that about five patients have to be treated to generate one positive outcome. One possible explanation for this finding is that the treatment of FoPS reduces generalized social anxiety, as an individual with generalized social anxiety has a performance anxiety (e.g., FoPS) in addition to an interaction anxiety (Blöte et al., 2009; Bögels et al., 2010). Such a perspective could also explain why the treatment effects of psychological interventions aimed at FoPS are lower for generalized social anxiety than for FoPS, as generalized social anxiety consists of more than a public speaking fear. Another possible interpretation is that the positive treatment effect on generalized social anxiety can be explained by the fact that questionnaires measuring generalized social anxiety also measure FoPS, to a certain extent.

It was also shown that psychological interventions aimed at FoPS for generalized social anxiety demonstrate a moderate to large (Hedges *g* = 0.70) effect size at follow-up. The effect size for generalized social anxiety at follow-up was thus larger than the same effect size at post-treatment. One possible explanation for this is that the effects not only remained at follow-up, but also increased with time. This is in accordance with a previous finding by Nordgreen et al. (2018). Following this explanation, it would seem that psychological interventions for FoPS continue to decrease generalized social anxiety symptoms over time. A possible interpretation of this is that reduced public speaking anxiety over time generalizes to fear reductions for other social situations. However, this difference might also reflect the different effect sizes utilized, where the post-treatment effect size was solely based on between-group comparisons, whereas the follow-up effect size was based on both between-group and within-group comparisons. As we could not control for these different methods in effect size calculation for the follow-up results on generalized social anxiety, this finding should be interpreted with caution.

#### Changes in Effect of Psychological Interventions After Treatment Termination

We conducted two additional meta-analyses to examine the effect of psychological interventions after treatment termination. This effect is commonly referred to as the “sleeper effect,” describing any type of delayed impact or effect on a recipient of an intervention after its termination (e.g., Flückiger et al., 2015).

The overall effect after treatment termination for psychological interventions aimed at FoPS from post-treatment to follow-up was small (*g* = 0.20), corresponding to an NNT of 8.89. Furthermore, the overall effect after treatment termination of psychological interventions on generalized social anxiety from post-treatment to follow-up was small (*g* = 0.23), corresponding to an NNT of 7.74. As heterogeneity was non-significant for both of these analyses, no meta-regression analyses were conducted in these cases.

Although the sleeper effect is commonly calculated by comparing changes between different therapeutic frameworks following treatment termination (Flückiger et al., 2015), we were precluded from this in the present meta-analysis. The reasons for this was the non-significant heterogeneity for both of the aforementioned meta-analyses, making meta-regression analyses redundant, and due to the fact that the studies that provided sufficient data for calculation of effect sizes after treatment termination all were cognitive and/or behavioral interventions. Consequently, only one group of therapeutic frameworks was available for these analyses. Still, our findings revealed an overall sleeper effect for cognitive and behavioral psychological interventions for FoPS as well as generalized social anxiety outcome measures. As all studies in these meta-analyses were cognitive or behavioral, one possible explanation for the continued effects after treatment termination might be that the therapies in question emphasize the importance for patients to utilize and continue to apply the techniques learned during treatment also after its termination.

#### Secondary Outcome Measures

As there were not enough studies with sufficient data, we could not pursue the final aim of this study and conduct any meta-analyses on our chosen secondary outcomes (i.e., depression, satisfaction with treatment, treatment expectancy, and treatment credibility).

### Limitations

The present meta-analysis has several limitations. One limitation is the risk of bias of included studies, with several studies not passing all four criteria in the applicable four domains of bias. The unknown risk of some of the included studies suggests our results should be interpreted with caution. However, we cannot conclude whether these studies are of actual high or low bias. This pertains in particular to the older studies included in the present review, due to poorer reporting of results in past decades. Another limitation of the present meta-analysis is that it did not include unpublished studies, as it has been argued by some authors (e.g., Cook et al., 1993) that such studies generally obtain lower effect sizes which in turn could potentially impact the effect sizes in meta-analyses. However, in their review of nearly 200 meta-analyses, Schmucker et al. (2017) argue that current empirical data demonstrates that the inclusion of unpublished studies rarely impacts the effect sizes in meta-analyses on a statistically significant level. Other recommended methods for assessing publication bias and its impact on effect sizes (e.g., Duval and Tweedie, 2000; Borenstein et al., 2009a; Schmucker et al., 2017), including examination of funnel plot symmetry and the adjustment of effect sizes using the Duval and Tweedie's trim and fill procedure were followed in the conduct of the present meta-analysis. Still, including unpublished data could potentially provide even more accurate estimates of effect sizes, making the omission of such unpublished data a limitation of the present study.

Another limitation of the present study is that the majority of the included studies were based on completers-only data. This may have led to an overestimation of effect sizes, perhaps due to the individuals not benefitting from the interventions dropping out or due to such incidents being unaccounted for in intention-to-treat analyses. Furthermore, our finding examining the differences between cognitive-behavioral interventions vs. other types of interventions should be interpreted with caution. Although our categorization of the cognitive-behavioral group was in accordance with previous research (Cuijpers et al., 2014), both groups compared in this analysis are quite heterogeneous. More research is therefore needed to examine the differences between therapeutic frameworks, as well as examining the effects of the interventions in the “other” category.

Although nearly none of the included studies reported researcher allegiance or therapist effects, such characteristics are important to examine as they can impact the effect size, a notion which is highlighted by the MAP-24 guidelines (Flückiger et al., 2018). There are examples of how indicators of such factors can be investigated in alternative ways (e.g., Del Re et al., 2012). Another limitation of the present study is therefore not accounting for these characteristics, which future reviews are encouraged to examine. Furthermore, future treatment studies should report data on researcher allegiance and therapist effects so that these variables can be investigated as potential moderators of treatment outcome in future meta-analyses. A general limitation with meta-analyses should also be considered, because such analyses provide an effect size estimate of how different psychological interventions compare to control groups. Thus, such a broad perspective comes at the cost of the detailed information on how the interventions work.

### Future Directions

It is noteworthy that the present meta-analysis did not examine whether interventions delivered through a group format, individualized format or self-help format differed in their ability to reduce symptoms of FoPS and generalized social anxiety. As previously mentioned, this was due to the fact that only a limited number of moderator variables could be selected in order to maintain a reasonable ratio of single effect sizes to the number of moderators. Future research is encouraged to attempt to clarify potential different outcomes for such formats.

Another important finding of the present review was that only one of the 30 included studies focused on the effects of psychological interventions on FoPS for adolescents. Because social anxiety and its associated forms, such as FoPS, have an onset during childhood and the adolescent years (American Psychiatric Association, 2013), it is essential for future studies to investigate the effects of psychological interventions on FoPS for this age group. It could be important to intervene early in an attempt to prevent the development of FoPS, but also because FoPS may significantly impair educational attainments (Van Ameringen et al., 2003), and furthermore may decrease the number of years an individual is impaired by FoPS. Moreover, early interventions on specific phobia such as FoPS can prevent the development of generalized social anxiety disorder (Wittchen and Fehm, 2003; Gregory et al., 2007; Blöte et al., 2009), making the treatment of FoPS highly important given the individual impairments and societal costs of social anxiety disorder. In this respect, an examination of how adolescents (and not only adults) respond to existing psychological interventions for FoPS may be of vital importance. With regards to future directions, we thus urge forthcoming studies to investigate the effects of psychological interventions for FoPS and social anxiety in adolescent populations. We would also like to direct researchers' attention to the lack of studies on FoPS and social anxiety in an elderly population, as noted elsewhere in the literature (Fehm et al., 2005).

Another important empirical implication of the present meta-analysis is that it directs forthcoming research to examine the effects of interventions for FoPS on relevant comorbidity. Given the strong association and the high comorbidity between social anxiety and depression (Kessler et al., 1999), it may be deemed an important additional task to assess the effects of the interventions toward FoPS on depression. Although lower in FoPS than social anxiety in its generalized form, the comorbidity between FoPS and major depressive disorder was found in one study to be about 2-fold, with an odds ratio of 2.1 (Stein and Chavira, 1998). It is therefore a surprising and important finding that few studies examining the effects of psychological interventions on FoPS have investigated its further effect on depressive symptoms.

Regarding the three other secondary outcomes that we could not examine due to insufficient data, it might be of interest for studies with newer interventions (e.g., technology-delivered interventions) to assess treatment credibility as well as the satisfaction with treatment. Furthermore, as treatment expectancy has been demonstrated to be an important predictor of psychotherapy outcome (Meyer et al., 2002; Greenberg et al., 2006), future studies should also investigate its role in the field of FoPS.

Additionally, more studies on FoPS reporting follow-up results that utilize other types of theoretical framework than cognitive and behavioral treatments are needed, as this would allow future meta-analyses to investigate the potential sleeper effects of these interventions. Finally, future studies should examine several potential moderators that could have an impact on the effect of psychological interventions for FoPS. More specifically, possible moderators include comorbidity, the utilization of manuals, treatment adherence, therapist effects, and researcher allegiance. It is important for future outcome studies to report such characteristics, so that they can be investigated as potential moderators in future meta-analyses.

## Conclusions

The present meta-analysis is of importance as it informs the treatment of FoPS, relieving its negative educational, social, and occupational consequences. It fills an important knowledge gap as it is the first meta-analysis on FoPS for three decades, and because it is the first meta-analysis to date to examine the effects of psychological interventions for FoPS exclusively based on RCTs. From this meta-analysis, it seems safe to conclude that psychological interventions aimed at FoPS are both effective for the FoPS as well as the more generalized form of social anxiety in an adult population. Furthermore, psychological interventions have beneficial long-term effects in the treatment of FoPS. Additionally, a sleeper effect was found for cognitive and behavioral interventions, indicating that patients receiving these interventions continued to improve after treatment termination. The effects of psychological interventions were robust against active control groups (e.g., attention placebo) as well as passive control groups (e.g., waiting list). With regard to the high prevalence, the impairments that FoPS and social anxiety have on functioning and well-being, as well as their societal costs, and the findings highlighting how FoPS in both adolescence and adulthood is associated with the development of generalized social anxiety disorder, we find these to be important conclusions, suggesting that psychological interventions are effective and are associated with moderate to large effects at post-treatment as well as large effects on follow-up for FoPS. Moreover, the present study found no difference between technology-delivered interventions (i.e., virtual reality and Internet-delivered interventions) and traditional interventions such as therapy delivered face-to-face for FoPS. This is an important finding, suggesting that treatment outcome is not dependent on mode of delivery, implying that clinicians can exert some flexibility in terms of the way treatment is administered. This finding further highlights an opportunity to increase access to evidence-based treatments through technology-delivered interventions, which can be implemented at schools, in primary care and specialist mental health care.

## Data Availability

The data supporting the conclusions of this manuscript will be made available by the authors, without undue reservation, to any qualified researcher. Requests to access the datasets should be directed to omid.ebrahimi@student.uib.no.

## Author Contributions

OE designed the study and wrote the protocol under the supervision of TN and RK. OE conducted the literature search. RK assisted OE in the reviewing process. OE and SP proceeded with the data extraction process. Statistical analyses and risk of bias assessment was conducted by OE under the supervision of SP. OE wrote the first draft of the manuscript. The manuscript continued its development under OE with involvement from TN, RK, and SP. All authors contributed to and have approved the final manuscript.

### Conflict of Interest Statement

The authors declare that the research was conducted in the absence of any commercial or financial relationships that could be construed as a potential conflict of interest.
