# Supplementary material for: Psychological Interventions for the Fear of Public Speaking: A Meta-Analysis
Source: Front Psychol. 2019 Mar 15;10:488. doi: 10.3389/fpsyg.2019.00488 (PMC6428748; doi:10.3389/fpsyg.2019.00488)
Supplement: Supplementary file 1 [file Table_1.doc]

**Supplemental Material A. Search Strategies**

| **Search terms and databases** |
| --- |
| Unless otherwise stated, search terms are free text terms. Abbreviations: ‘adj’: adjacent (i.e. number of words within range of search term); ‘near/x’: finds the terms when they are within x words of each other where x = the maximum number of words between search terms and where terms can appear in either order; ‘next’: finds the terms when they appear next to each other; ‘ti’: title; ‘ab’: abstract; ‘kw’: keyword where limit includes MeSH terms but does not allow for MeSH term explosion; ’‘/’: represents Subject Headings in the PsycINFO database; ‘mp’ in the MedLine database includes: title, abstract, original title, name of substance word, subject heading word, keyword heading word, protocol supplementary concept word, rare disease supplementary concept word, unique identifier, synonyms; ‘mp’ in the PsycINFO database includes: title, abstract, heading word, table of contents, key concepts, original title, tests & measures; MeSH: medical subject heading (MEDLINE and Cochrane Library medical index term); ‘SCI-Expanded’: Science Citation Index Expanded; ‘SSCI’: Social Science Citation Index; ‘A&HCI’: Arts and Humanities Citation Index; ‘ESCI’: Emerging Sources Citation Index |
| ***The Cochrane Library*** |
| 1 (("public-speaking" or "speaking in public") near/2 ("anxi*" or "fear*" or "phobi*")):ti,ab,kw (Word variations have been searched)  2 ("speech anxiety" or "speech phobia"):ti,ab,kw (Word variations have been searched)  3 ("presentat*" next ("anxiety" or "phobi*" or "fear*")):ti,ab,kw (Word variations have been searched)  4 ("communication apprehension" or "public-speaking apprehension" or "public speaking communication apprehension"):ti,ab,kw (Word variations have been searched)  5 #1 or #2 or #3 or #4  6 ("treatment*" or "therap*" or "intervention*" or "psychotherap*"):ti,ab,kw (Word variations have been searched)  7 #5 and #6 |
| **MEDLINE (Ovid)** |
| 1 (("public-speaking" or "speaking in public") adj3 ("anxi*" or "fear*" or "phobi*")).mp  2 ("speech anxiety" or "speech phobia").mp  3 ("presentat*" adj ("anxiety" or "phobi*" or "fear*")).mp  4 "fear of presenting".mp  5 ("communication apprehension" or "public-speaking apprehension" or "public speaking communication apprehension").mp  6 1 or 2 or 3 or 4 or 5  7 ("treatment*" or "therap*" or "intervention*" or "psychotherap*").mp  8 7 and 6 |
| **PsycINFO (OvidSP)** |
| 1 (("public-speaking" or "speaking in public") adj3 ("anxi*" or "fear*" or "phobi*")).mp  2 ("speech anxiety" or "speech phobia").mp  3 ("presentat*" adj ("anxiety" or "phobi*" or "fear*")).mp  4 "fear of presenting".mp  5 speech anxiety/  6 ("communication apprehension" or "public-speaking apprehension" or "public speaking communication apprehension").mp.  7 1 or 2 or 3 or 4 or 5 or 6  8 ("treatment*" or "therap*" or "intervention*" or "psychotherap*").mp  9 7 and 8 |
| **Web of Science** |
| 1 ((("public-speaking" or "speaking in public") NEAR/2 ("anxi*" or "fear*" or "phobi*"))) Indexes=SCI-EXPANDED, SSCI, A&HCI, ESCI Timespan=All years  2 (("speech anxiety" or "speech phobia")) Indexes=SCI-EXPANDED, SSCI, A&HCI, ESCI Timespan=All years  3 (("presentat*" NEAR/0 ("anxiety" or "phobi*" or "fear*"))) Indexes=SCI-EXPANDED, SSCI, A&HCI, ESCI Timespan=All years  4 (("communication apprehension" OR "public-speaking apprehension" OR "public speaking communication apprehension"))  Indexes=SCI-EXPANDED, SSCI, A&HCI, ESCI Timespan=All years  5 #4 or #3 OR #2 OR #1 Indexes=SCI-EXPANDED, SSCI, A&HCI, ESCI Timespan=All years  6 (("treatment*" or "therap*" or "intervention*" or "psychotherap*")) Indexes=SCI-EXPANDED, SSCI, A&HCI, ESCI Timespan=All years  7 #6 AND #5 Indexes=SCI-EXPANDED, SSCI, A&HCI, ESCI Timespan=All years |
|  |

**Additional information**

“fear of presenting” resulted in 0 hits in Web of Science and was therefore removed from the search string.

“counselling” (more specifically “counsel*”) was also tested as a keyword, not yielding any additional results, therefore being removed from the search string.

Other variations of the words related to public speaking anxiety, public speaking and variations of fear of presenting/presentations were also tested not resulting in any new relevant hits.
