# Supplementary figures and images for: Psychological Interventions for the Fear of Public Speaking: A Meta-Analysis
Source: Front Psychol. 2019 Mar 15;10:488. doi: 10.3389/fpsyg.2019.00488 (PMC6428748; doi:10.3389/fpsyg.2019.00488)

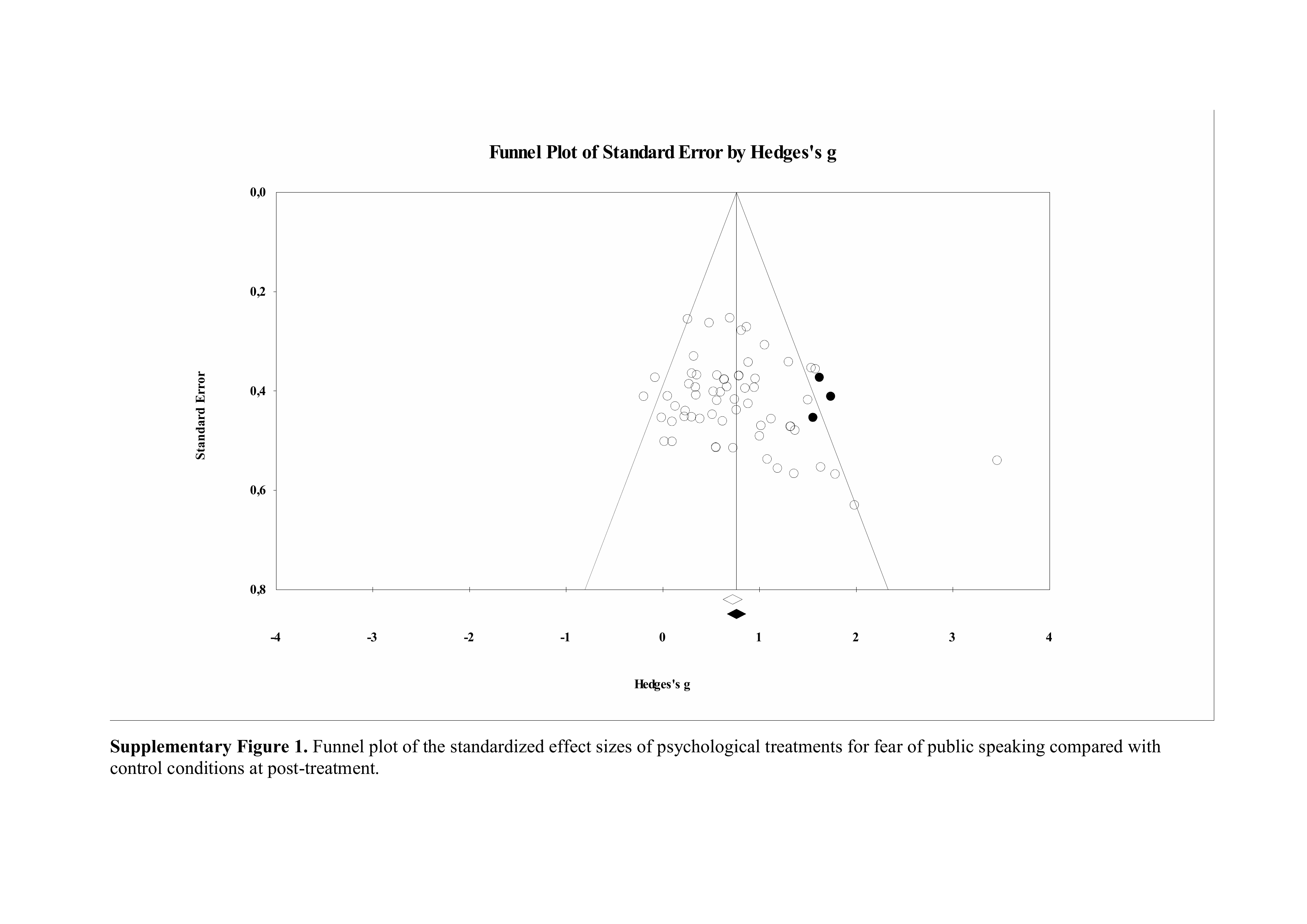

Supplement: Supplementary file 2 [file Image_1.TIF]

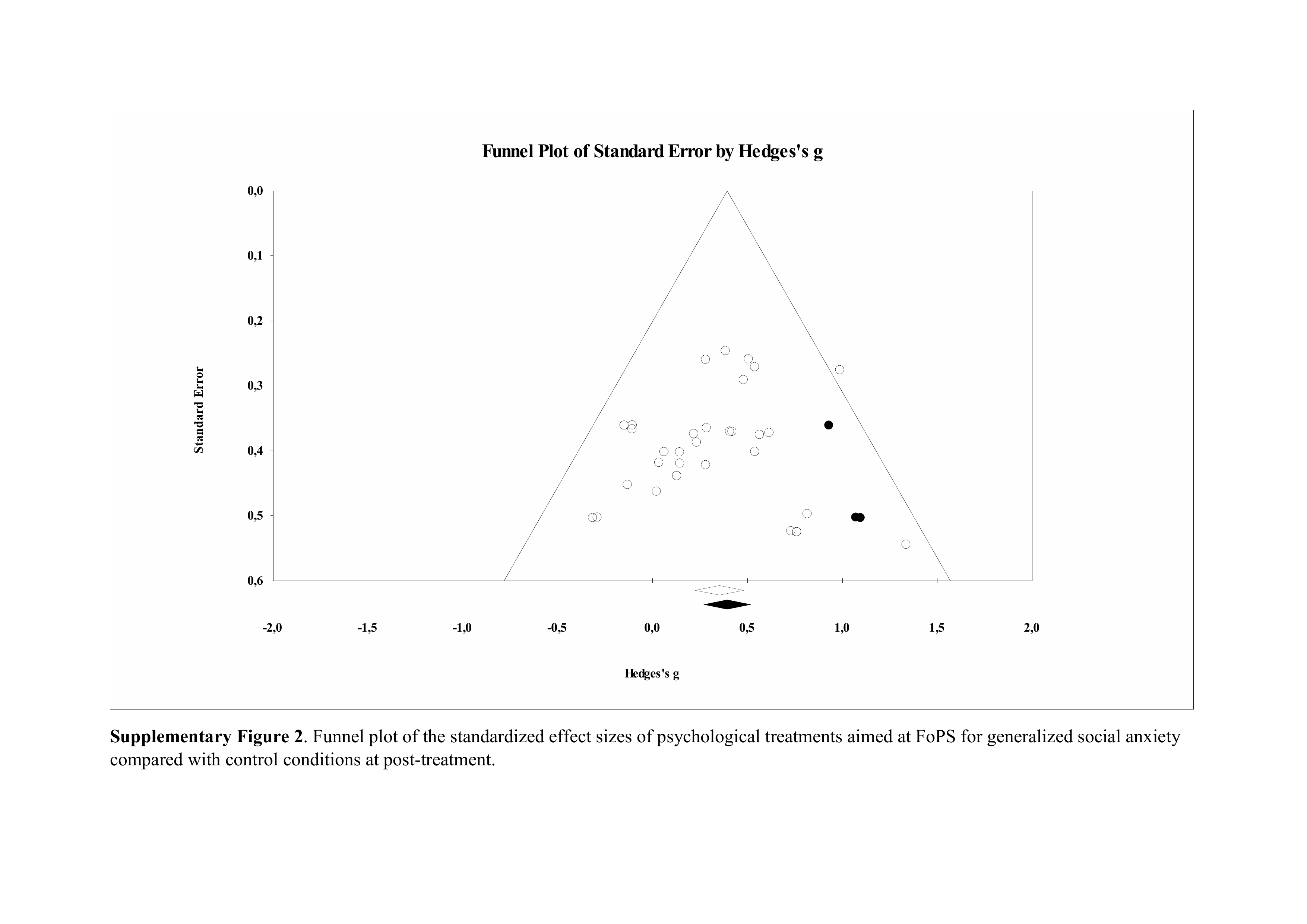

Supplement: Supplementary file 3 [file Image_2.TIF]

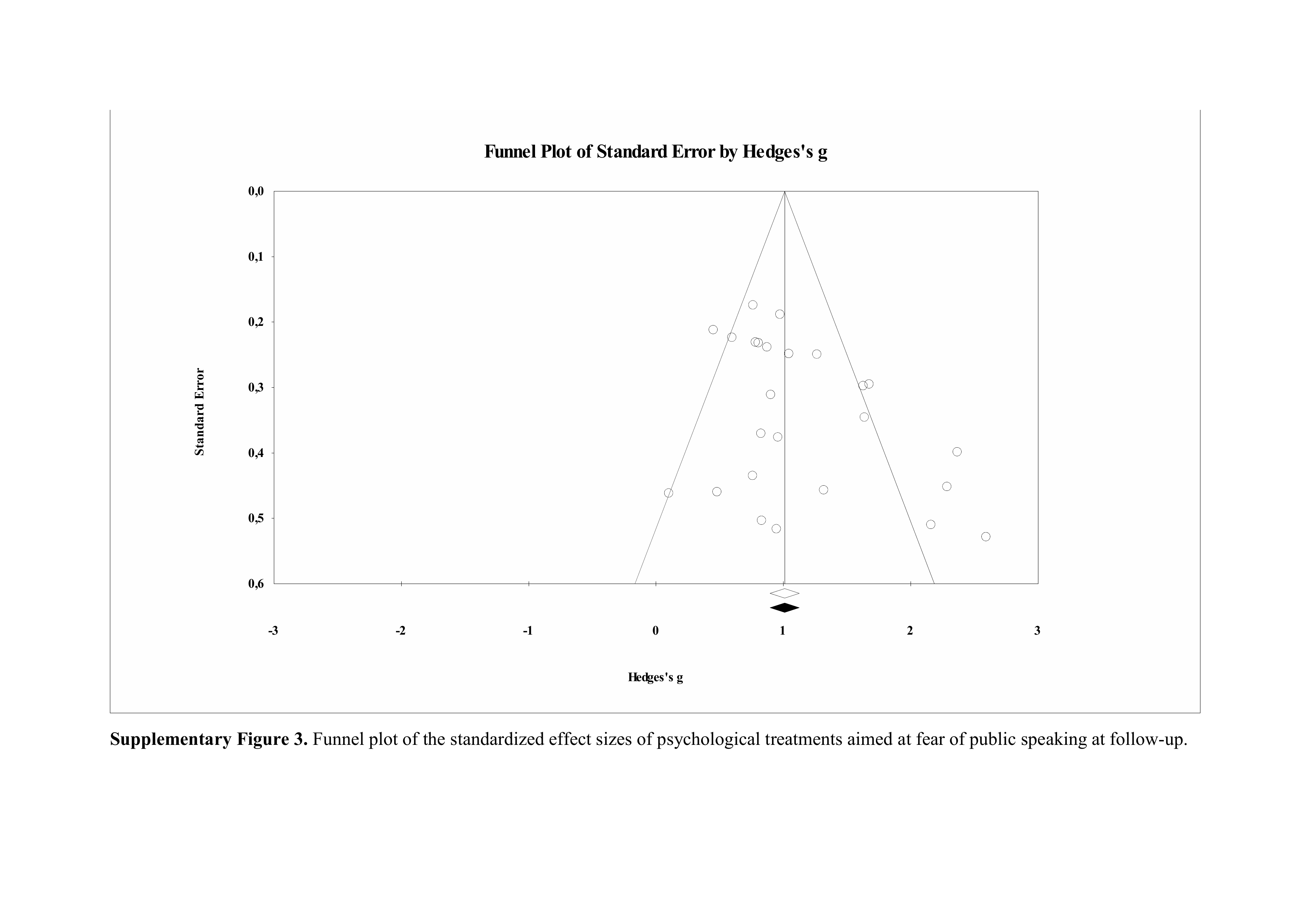

Supplement: Supplementary file 4 [file Image_3.TIF]

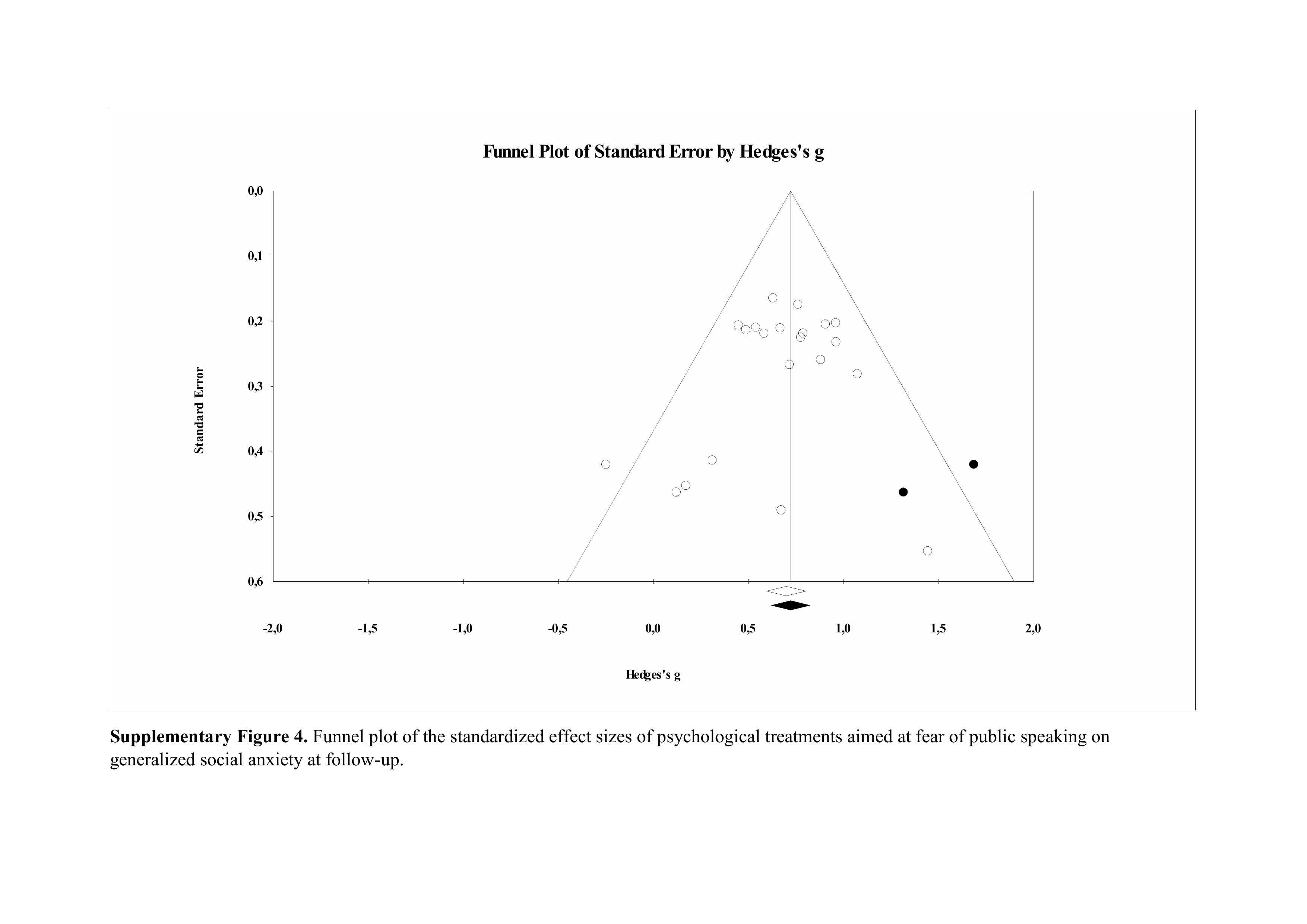

Supplement: Supplementary file 5 [file Image_4.TIF]

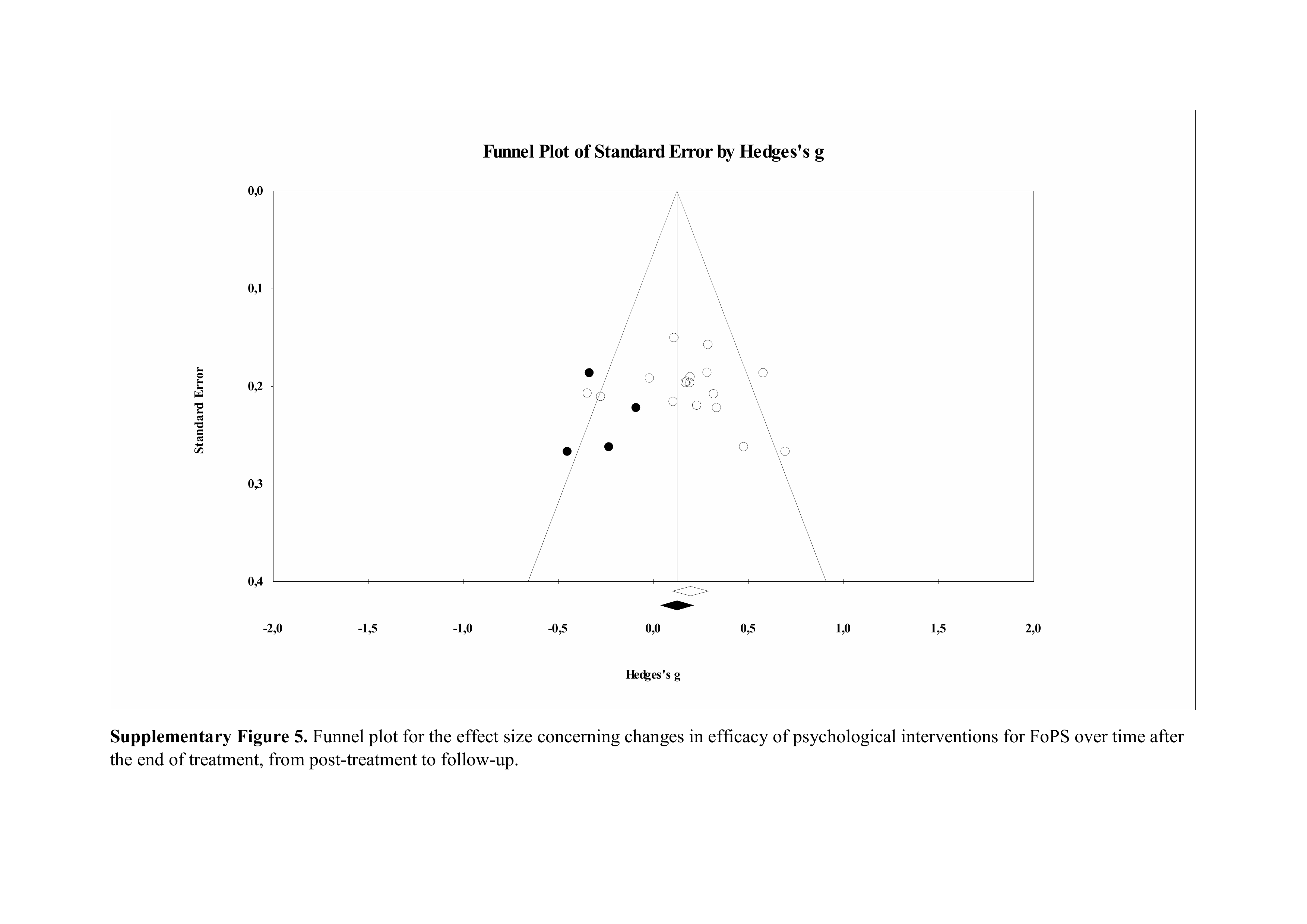

Supplement: Supplementary file 6 [file Image_5.TIF]

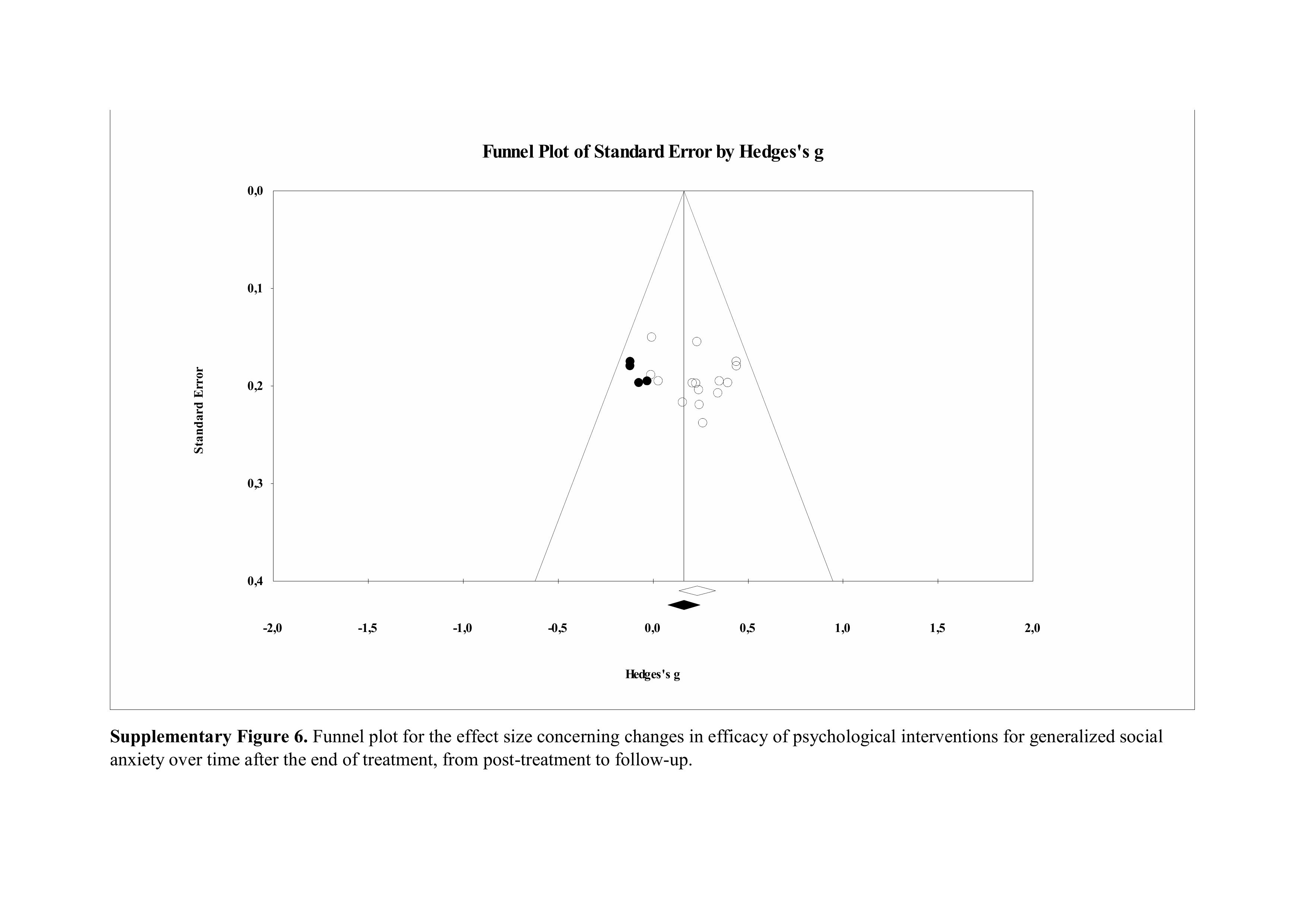

Supplement: Supplementary file 7 [file Image_6.TIF]
